# When and where can coastal wetland restoration increase carbon sequestration as a natural climate solution?

**DOI:** 10.1017/cft.2024.14

**Published:** 2024-10-11

**Authors:** Scott F. Jones, Ariane Arias-Ortiz, Dennis Baldocchi, Meagan Eagle, Daniel A. Friess, Catrina Gore, Greg Noe, Stefanie Nolte, Patty Oikawa, Adina Paytan, Jacqueline L. Raw, Brian J. Roberts, Kerrylee Rogers, Charles Schutte, Camille L. Stagg, Karen M. Thorne, Eric J. Ward, Lisamarie Windham-Myers, Erik S. Yando

**Affiliations:** 1 Department of Biology, University of North Florida, Jacksonville, FL, USA; 2 Physics Department, Universitat Autònoma de Barcelona, Barcelona, Spain; 3 Department of Environmental Science, Policy, and Management, University of California, Berkeley, Berkeley, CA, USA; 4 Woods Hole Coastal and Marine Science Center, U.S. Geological Survey, Woods Hole, MA, USA; 5 Department of Earth & Environmental Sciences, Tulane University, New Orleans, LA, USA; 6 School of Environmental Sciences, University of East Anglia, Norwich, UK; 7 Florence Bascom Geoscience Center, U.S. Geological Survey, Reston, VA, USA; 8 Centre for Environment, Fisheries and Aquaculture Science, Lowestoft, UK; 9 Department of Earth & Environmental Sciences, California State University, East Bay, Hayward, CA, USA; 10 Department of Earth and Planetary Science, University of California, Santa Cruz, Santa Cruz, CA, USA; 11 Anthesis, Cape Town, South Africa; 12 Institute for Coastal and Marine Research, Nelson Mandela University, Gqeberha, South Africa; 13 Louisiana Universities Marine Consortium, Chauvin, LA, USA; 14 School of Earth, Atmospheric and Life Sciences, University of Wollongong, Wollongong, NSW, Australia; 15 Department of Environmental Science, Rowan University, Glassboro, NJ, USA; 16 Wetland and Aquatic Research Center, U.S. Geological Survey, Lafayette, LA, USA; 17 Western Ecological Research Center, U.S. Geological Survey, Davis, CA, USA; 18 Earth System Science Interdisciplinary Center, University of Maryland, College Park, MD, USA; 19 Biospheric Science Laboratory, NASA Goddard Space Flight Center, Greenbelt, MD, USA; 20 Water Resources Mission Area, U.S. Geological Survey, Menlo Park, CA, USA; 21 Department of Biological Sciences, Old Dominion University, Norfolk, VA, USA

**Keywords:** natural climate solutions, coastal wetlands, ecosystem management, restoration, carbon sequestration

## Abstract

Coastal wetlands are hotspots of carbon sequestration, and their conservation and restoration can help to mitigate climate change. However, there remains uncertainty on when and where coastal wetland restoration can most effectively act as natural climate solutions (NCS). Here, we synthesize current understanding to illustrate the requirements for coastal wetland restoration to benefit climate, and discuss potential paths forward that address key uncertainties impeding implementation. To be effective as NCS, coastal wetland restoration projects will accrue climate cooling benefits that would not occur without management action (additionality), will be implementable (feasibility) and will persist over management-relevant timeframes (permanence). Several issues add uncertainty to understanding if these minimum requirements are met. First, coastal wetlands serve as both a landscape source and sink of carbon for other habitats, increasing uncertainty in additionality. Second, coastal wetlands can potentially migrate outside of project footprints as they respond to sea-level rise, increasing uncertainty in permanence. To address these first two issues, a system-wide approach may be necessary, rather than basing cooling benefits only on changes that occur within project boundaries. Third, the need for NCS to function over management-relevant decadal timescales means methane responses may be necessary to include in coastal wetland restoration planning and monitoring. Finally, there is uncertainty on how much data are required to justify restoration action. We summarize the minimum data required to make a binary decision on whether there is a net cooling benefit from a management action, noting that these data are more readily available than the data required to quantify the magnitude of cooling benefits for carbon crediting purposes. By reducing uncertainty, coastal wetland restoration can be implemented at the scale required to significantly contribute to addressing the current climate crisis.

## Graphical abstract

**Figure:**
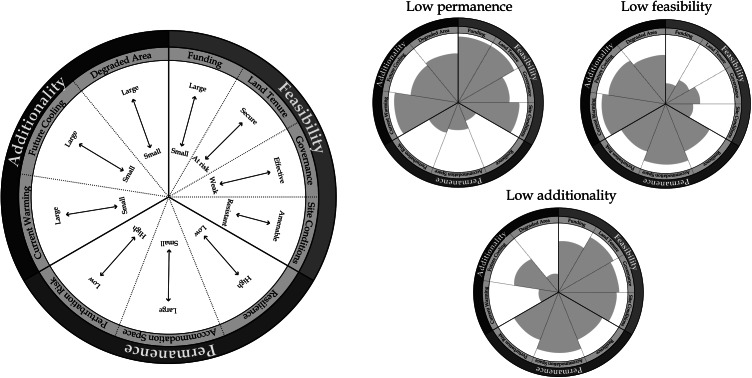
**Graphical Abstract**. Conceptual model for visualizing the three fundamental requirements for when and where coastal wetland restoration is effective as a natural climate solution (NCS): additionality, feasibility and permanence. Within each requirement, rays are the specific values that maximize effectiveness; values toward the outside of the circle are ideal (key to values inside each ray). Three example scenarios are given where restoration may not be effective as NCS but may still provide other co-benefits. Low permanence: for example, a site that experiences constrained topography, high rates of relative sea-level rise and/or low resilience. Low feasibility: for example, a site where land ownership and tenure are contentious, funding is not secured for restoration activities and/or existing site conditions preclude effective restoration. Low additionality: for example, a region with little area of degraded habitat, alternative land uses are not strong carbon sources and/or natural ecosystems are not strong carbon sinks (conservation may be more appropriate in this instance).

## Impact statement

Coastal wetlands, including mangrove forests, tidal marshes and seagrass meadows, can take carbon out of the atmosphere and store it in plant tissue and soil at the highest rates of any ecosystem. Because of this unique feature, coastal wetland restoration can act as a natural climate solution (NCS), helping to mitigate climate change by having a net cooling benefit compared to pre-restoration conditions. However, uncertainty remains in when and where coastal wetland restoration acts as effective NCS. This manuscript synthesizes the fundamental requirements for restoration to act as effective NCS: additionality, permanence and feasibility. We highlight the minimum data required to understand these requirements, which are less robust than the data needed for carbon crediting or accounting. Many of these data are spatial and widely available. We also highlight future perspectives that may help address uncertainty in restoration as NCS, by taking a landscape-scale approach and incorporating methane emissions. Ultimately, reducing uncertainty in when and where coastal wetland restoration acts as NCS supports the broader effort to mitigate climate change most effectively.

## Coastal wetlands as natural climate solutions

Climate change is causing cascading impacts to human and natural systems globally, and all possible mitigation and adaptation actions will be needed to keep warming below critical thresholds over the next decade (United Nations Framework Commission on Climate Change (UNFCCC), 2015; Intergovernmental Panel on Climate Change (IPCC), 2022; Diffenbaugh and Barnes, 2023). For coastal landscapes, sea-level rise is among the greatest drivers of change, impacting coastal communities through increased flooding and salinization risks (Intergovernmental Panel on Climate Change (IPCC), 2021; Sweet et al., 2022). Natural climate solutions (NCS), or those actions that mitigate climate change using ecosystem management, can remove greenhouse gases from the atmosphere, complementing efforts to reduce fossil fuel emissions (Fargione et al., 2018; Macreadie et al., 2021; United Nations Environment Programme (UNEP) and International Union for Conservation of Nature (IUCN), 2021). Although we explicitly focus on NCS as actions that remove greenhouse gases here (without concurrent negative impacts; Ellis et al., 2024), restoration of coastal ecosystems comes with a host of additional co-benefits (Hagger et al., 2022; Krauss et al., 2022a; Rogers et al., 2023b; Novick et al., 2024).

**Figure 1.**
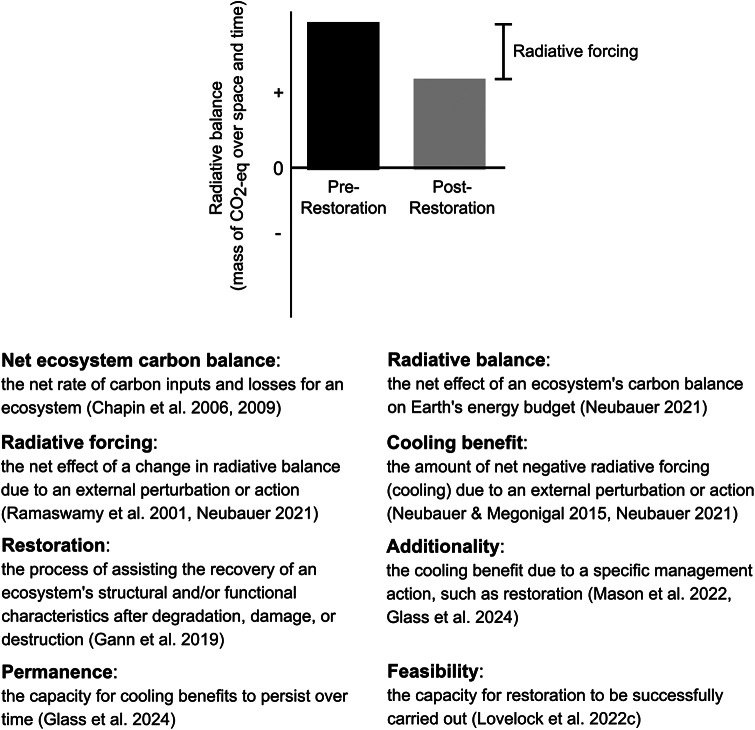
Key terms as defined in this manuscript. Conceptual comparison is of the radiative balance of a coastal wetland in pre-restored (black) and post-restored (gray) states (modified from Neubauer, 2021). In this example, the pre-restored and post-restored states both have positive radiative balances, adding energy to Earth’s energy budget. After restoration, there is a change in radiative balance (i.e., a radiative forcing); restoration action led to a reduction in radiative balance. Because the radiative forcing is negative, this example indicates a cooling benefit from restoration actions; the project has additionality.

Coastal wetlands, including mangrove forests, tidal marshes and seagrass meadows (among all other tidal wetlands; Adame et al., 2024), are highly productive ‘blue carbon’ ecosystems connecting terrestrial and marine realms globally. These ecosystems are unique in their ability to mitigate climate change as they continually absorb and store carbon from the atmosphere, leading to a climate cooling benefit (Figure 1; Neubauer, 2021) that grows over time if they continue to add carbon within the accommodation space created by sea-level rise (Rogers et al., 2019a; Buffington et al., 2021). Present day coastal wetlands initiated development when relative sea-level rise decelerated sufficiently for coastal wetlands to maintain their position within the tidal frame; the timing of this development varies globally due to differences in glacio-isostatic adjustment of coastlines (Woodroffe, 2019). Global distribution of blue carbon ecosystems is variable as well and largely determined by climate constraints (McKenzie et al., 2020; Jia et al., 2023; Worthington et al., 2024); these ecosystem types vary in how they store and cycle carbon to mitigate climate change.

At the regional scale, hydrogeomorphic setting (i.e., landscape configuration) constrains the occurrence of blue carbon ecosystem types and their ability to store carbon. Hydrogeomorphic setting influences the dominance of water forcings (e.g., wind, wave, tide; Boyd et al., 1992), sediment availability and deposition (Hupp et al., 2019), connectivity to other habitats (Noe et al., 2016; Woo et al., 2022), and freshwater availability and timing important for sulfate concentrations and methane production (Poffenbarger et al., 2011; Knox et al., 2021). As one example, intermittently connected lakes and lagoons (ICOLLs) or temporarily open/closed estuaries (TOCEs), are coastal wetlands that can undergo state shifts in salinity and water level that drive changes in ecosystem parameters like macrophyte community extent and composition (Riddin and Adams, 2008), presenting specific challenges in quantifying dynamic climate benefits.

Accounting for the temporal evolution of coastal wetlands can be challenging for practitioners, researchers and policy-makers alike (Neubauer and Megonigal, 2015; Neubauer, 2021; Abernethy and Jackson, 2022). Continuous and effectively permanent soil carbon sequestration, a particularly important aspect of coastal wetlands as blue carbon ecosystems, is a long-term additive process (Chmura et al., 2003; Mcleod et al., 2011). In the context of NCS, however, decadal timescales are of primary interest to assist in meeting climate commitments as soon as possible (United Nations Framework Commission on Climate Change (UNFCCC), 2015; United Nations Environment Programme (UNEP) and International Union for Conservation of Nature (IUCN), 2021). Greenhouse gas fluxes and herbaceous biomass can respond rapidly to management actions in coastal wetlands (Wang et al., 2021; Woo et al., 2022), and carbon sequestration rates and woody biomass can also recover within decades in certain situations (Marbà et al., 2015; Osland et al., 2020; Eagle et al., 2022; Rogers et al., 2023a). Regardless, losing millennia of stored carbon simply cannot be regained over short timescales by restoration; preservation of existing carbon stocks and functioning ecosystems is therefore key (Drexler et al., 2009; Arias-Ortiz et al., 2021a).

Given historical degradation and land conversion of coastal wetlands globally (Friess et al., 2019; Turschwell et al., 2021; Campbell et al., 2022), under-recognized but tractable opportunities exist to use restoration as NCS to recover carbon sequestration functionality (Macreadie et al., 2017; United Nations Environment Programme (UNEP) and International Union for Conservation of Nature (IUCN), 2021; Krauss et al., 2022b; Lovelock et al., 2022a). Hydrologic impoundment is a leading cause of stress and degradation for intertidal coastal ecosystems (Montague et al., 1987; Warren et al., 2002; Lewis et al., 2016; Chambers et al., 2019). Reconnecting degraded wetlands to their watersheds is therefore a common restoration technique, with documented success in halting oxidative loss of carbon stores or otherwise shifting carbon cycling for a climate cooling benefit (Kroeger et al., 2017; Dittmann et al., 2019; Cormier et al., 2022; Eagle et al., 2022; Windham-Myers et al., 2023). Sediment augmentation is also commonly used to increase resilience to relative sea-level rise in coastal wetlands that have deteriorated from increased flooding stress (Stagg and Mendelssohn, 2010; Yuan et al., 2022; Fard et al., 2024), leading to enhanced longevity of carbon sequestration compared to no-action alternatives. Additionally, improving water quality (e.g., eutrophication) and other threats (Turschwell et al., 2021) before introducing large numbers of foundation species may be critical for seagrass restoration success (van Katwijk et al., 2016).

Regardless of restoration approach, coastal wetlands have been identified as particularly impactful habitats for restoration actions as NCS because of (a) their high rates of carbon sequestration and high densities of carbon storage over centuries to millennia (Bridgham et al., 2006; Mcleod et al., 2011; Poulter et al., 2022); (b) the potential for management actions that have meaningful impacts on carbon budgets of degraded habitats, leading to climate cooling benefits; and (c) the potential for interventions to have additional social and environmental co-benefits (Lovelock and Duarte, 2019). Given that opportunities for restoration are distributed unevenly across continental scales (e.g., Holmquist et al., 2023) and resources for restoration activity are limited, there remains a lack of clarity on where coastal wetland restoration is maximally effective as NCS, and under which circumstances action is warranted.

Ultimately, to be effective as NCS, coastal wetland restoration projects must accrue climate cooling benefits that would not occur without management action (Figure 1). Here, we synthesize current understanding to 1) illustrate the fundamental requirements for coastal wetland restoration to be an effective NCS, addressing uncertainty in where restoration maximizes climate benefits, and 2) discuss potential paths forward to overcome current implementation barriers, addressing uncertainty in when restoration action is warranted.

## Requirements for coastal wetland restoration as an effective natural climate solution

Three fundamental criteria determine the effectiveness of restoration actions as NCS: additionality, feasibility and permanence (Table 1). Below, we discuss requirements in an ecological sense, rather than within the context of a particular carbon finance or accounting framework. Due to their potential to influence site-specific climate benefits, local-scale factors are also considered.

**Table 1.** The fundamental requirements for coastal wetland restoration to be effective as NCS: additionality, feasibility and permanence

Requirements	Values that maximize climate benefit	Minimum data required to quantify
Additionality	Current pre–restoration conditions contribute to substantial climate warming (large positive radiative balance) Expected post–restoration conditions contribute to substantial climate cooling (large negative radiative balance), or contribute to substantially less climate warming in comparison to pre–restoration conditions Large area of degraded, restorable habitat is available in the region Potential for biomass carbon gain is high, particularly of woody plant species	Regional land use/land cover maps at a sufficient resolution and specificity to allow area calculations for each land use/land cover class Regional carbon radiative balance or emissions/removal factor estimates by land use/land cover classes
Feasibility	Funding is secured and appropriate in scale Land tenure is secured, clearly communicated and respected Local communities are part of the project team and will gain access to co–benefits of restoration for NCS Governance is effective in the region Pre–restoration biophysical conditions are amenable to regional restoration practices/culture	Regional land ownership/tenure maps at a sufficient resolution to allow project planning Estimates of restoration cost per area restored given regional restoration culture and available financial incentives Local reports of existing communication and collaboration among local communities to understand end–user involvement and investment
Permanence	High capacity for resilience through allochthonous processes (e.g., large sediment supply) High capacity for resilience through autochthonous processes (e.g., high rates of root production) Upslope and/or upstream accommodation space is available and accessible for wetland migration Low risk of short–term perturbations	Regional estimates of coastal wetland resilience to relative sea–level rise Regional topography maps, including anthropogenic alterations to topography that influence hydrology, at a sufficient resolution to allow project planning Local reports from communities on equitable distribution of restoration co–benefits

*Note*: Specific values of these requirements can maximize the cooling benefit of coastal wetland restoration. There are relatively straightforward minimum data needed to quantify if the fundamental requirements are met to address the question, ‘Does this management action lead to a net climate benefit?’

### Additionality

Coastal wetland restoration is effective as NCS when actions ‘add’ carbon to the landscape, reducing atmospheric greenhouse gas concentrations and leading to a cooling benefit compared to initial degraded conditions (Figure 1). Maximal cooling benefits occur where the difference in pre-restoration and post-restoration climate impact is large. For example, highly degraded pre-restoration sites with large carbon emissions being converted to productive post-restoration sites with large carbon sequestration maximizes additionality. This cooling benefit can be achieved through restoring areas back to their original ecosystem type (e.g., conversion of shrimp ponds back to mangrove forests; Sidik et al., 2019), enhancing or rehabilitating function within an ecosystem type (e.g., restoring hydrology to impounded marshes, Eagle et al., 2022), or creating new/novel habitat. Large areas available in degraded condition that can be converted through management action to an enhanced condition equates to large potential cooling benefits. Small estuarine systems therefore may not have the same potential as large deltas/bays (unless aggregated as regional systems; Duarte de Paula Costa et al., 2022), because habitat size (i.e., degraded land that can be restored) was originally small. Beyond size considerations, often ignored but potentially important biophysical changes can occur after restoration, leading to net cooling benefits without changing carbon cycling directly (e.g., changes in albedo, latent/sensible heat flux, roughness; Graf et al., 2023; Zhu et al., 2024).

Conditions amenable to quick recovery of carbon storage pools, reduction in greenhouse gas emissions, and/or enhanced carbon sequestration rates are key to maximizing additionality in coastal wetland restoration. While most carbon is stored in coastal wetland soils over the long-term, biomass pools often develop more rapidly and can be the first sign of additionality from restoration (Rogers et al., 2023a). Habitat types with large woody vegetation (characteristic of mangrove and tidal forests) contain substantially more biomass carbon than habitat types with herbaceous vegetation (characteristic of tidal marshes and seagrasses) (Adame et al., 2024), and can amass considerable additionality over 15–25 years after restoration (Osland et al., 2020; Rogers et al., 2023a). Restored sites that have the potential for large gains in biomass carbon after management action may therefore maximize additionality over decadal scales (e.g., Sasmito et al., 2019). This additional vegetation biomass can be constrained by regional scale factors (e.g., Rovai et al., 2021 for mangroves). The accommodation space for carbon burial in an estuary also varies regionally, based largely on geologic ‘maturation’ stage (Owers et al., 2022; Rogers et al., 2022). Regionally variable sediment availability for allochthonous carbon burial and freshwater availability to support autochthonous production can drive the potential for adding carbon to the landscape as well (e.g., Thorne et al., 2022) (see the discussion on allochthonous carbon in Section ‘Coastal wetlands as cross-ecosystem linkages’). Additionality after restoration may not follow a linear increase, instead showing rapid initial responses (e.g., for carbon accumulation; Burden et al., 2019). For effective cooling, additionality and general carbon cycling after restoration do not need to match remnant ecosystems; there needs to be enhanced function compared to the initial/alternative degraded state.

### Feasibility

Coastal wetland restoration is effective as NCS when actions are feasible to implement. Pinpointing areas on the landscape where restoration actions will have the largest benefits to climate mitigation is inconsequential if the actions themselves cannot be completed. Feasibility is largely set by conditions external to the restoration site, including regional socioeconomic and governance constraints that influence human decision-making (Friess et al., 2019; Stewart-Sinclair et al., 2020). Restoration can take considerable infrastructure and funding to implement; this funding must be in place or accessible in the region for action to commence, and may use a variety of financial instruments (Friess et al., 2022). Regional and local land tenure is an additional crucial consideration for effective restoration (Lovelock and Brown, 2019; Lovelock et al., 2022c; Bell-James et al., 2023), as additional co-benefits should be delivered to local communities and stakeholders, who are often direct (and historical) users of coastal ecosystems (Wylie et al., 2016; Dencer-Brown et al., 2022). Existing policies and regulations can vary in scope and purpose across jurisdictional lines, making a complex web that may impede effective coastal management, including restoration activities (Herr et al., 2019). To maximize feasibility, external conditions will support restoration action through available funding, appropriate land tenure, and effective governance (Stewart-Sinclair et al., 2020; Macreadie et al., 2022; Windham-Myers et al., 2023).

Maximizing feasibility also includes ensuring internal site conditions are amenable to region-specific restoration culture and practice. Pre-restoration land use can influence post-restoration vegetation and water quality recovery, through impacts on elevation and initial plant community composition (Janousek et al., 2020). Restoration activities can also fail when restoration practice does not align with local site conditions. For instance, planting mangrove propagules on mudflats for rehabilitation can have low survival rates if species are used that are unlikely to naturally establish at available elevations (Wodehouse and Rayment, 2019; Lovelock et al., 2022c). Further, the cultural practice of restoration itself, including methods, goals, and rationale, may vary by region (e.g., Hudson and Kenworthy, 2021; Lovelock et al., 2022b) (Table 2). Feasible restoration actions mesh with the regional context of restoration practice and are therefore context specific; creating shared goals across diverse stakeholders can underpin feasibility and successful implementation in this regard (Surgeon Rogers et al., 2019).

**Table 2.** A non-exhaustive list of example methods and applicable case studies for restoration of coastal wetlands that may lead to climate cooling benefits

Restoration method	Description	Case study citations
(Un)Managed realignment	A type of hydrologic restoration. Breaching coastal embankments that were installed to convert historical wetlands to other land uses, either intentionally (managed) or unintentionally (unmanaged).	Masselink et al., 2017; Hudson and Kenworthy, 2021; Mossman et al., 2022
Regulated tidal exchange	A type of hydrologic restoration. Increasing tidal flow through engineered structures or managing estuary mouths in a controlled fashion.	Kroeger et al., 2017; Hudson and Kenworthy, 2021; Claasens et al., 2022; Cormier et al., 2022; Eagle et al., 2022
Subsidence reversal	A type of hydrologic restoration. Rewetting historical wetlands that have been converted to other drained land uses to halt and reverse subsidence processes.	Miller and Fujii, 2011; Windham–Myers et al., 2023
Re–seeding	A type of biotic restoration. Collecting and deploying seeds/propagules to restore foundation plant species populations.	Broome et al., 1988; Orth et al., 2012; Gamble et al., 2021; Sinclair et al., 2021
Re–planting	A type of biotic restoration. Collecting and transplanting shoots/seedlings to restore foundation plant species populations.	Broome et al., 1988; O’Brien and Zedler, 2006; Zamith and Scarano, 2010; Gamble et al., 2021; Sinclair et al., 2021; van Bijsterveldt et al., 2022
Sediment augmentation/placement	A type of geophysical restoration. Applying sediment to increase the elevation capital of a coastal wetland. When placement is from dredged material, can be called beneficial use.	Ray, 2007; Manning et al., 2021; Fard et al., 2024

(Broome et al., 1988; O’Brien and Zedler, 2006; Ray, 2007; Zamith and Scarano, 2010; Miller and Fujii, 2011; Orth et al., 2012; Kroeger et al., 2017; Masselink et al., 2017; Gamble et al., 2021; Hudson and Kenworthy, 2021; Manning et al., 2021; Sinclair et al., 2021; Claasens et al., 2022; Cormier et al., 2022; Eagle et al., 2022; Mossman et al., 2022; van Bijsterveldt et al., 2022; Windham-Myers et al., 2023; Fard et al., 2024)

### Permanence

Coastal wetland restoration is effective as NCS when cooling benefits are ‘permanent’ over management-relevant timescales. These timescales should be explicitly defined. Here, we propose decades are the appropriate permanence timescale, to align with 2050 emissions reduction targets (United Nations Framework Commission on Climate Change (UNFCCC), 2015; Intergovernmental Panel on Climate Change (IPCC), 2022). Permanence over management-relevant timescales occurs when restored sites are resilient to relative sea-level rise over the next several decades. Regional controls on sediment type and availability influence the capacity for wetland vertical accretion of allochthonous material, and therefore resilience (Rovai et al., 2018; Gorham et al., 2021; Breithaupt and Steinmuller, 2022). Resilient restoration will balance rates of relative sea-level rise and sediment supply to be successful; restoration at low elevation sites where sediment supply is low risks failure as vegetation may be rapidly overwhelmed by rising sea levels or erosion from wave action. However, where sediment supply is ample, restoration at lower elevations may still be successful as rapid gains in elevation and carbon addition from root biomass may occur (Liu et al., 2021; Mossman et al., 2022). Permanence can also occur where autochthonous production is high, particularly in more biogenic/organogenic settings (Krauss et al., 2017; Cahoon et al., 2021; Windham-Myers et al., 2023). Restored coastal wetlands do not need to depend on vertical processes alone for decadal-scale permanence. Where geomorphic development has led to available accommodation space and land use is amenable, lateral migration into upland or upstream habitats can allow continued cooling benefits of coastal wetland restoration activity even where vertical elevation-building processes are expected to be overwhelmed (Osland et al., 2022; Owers et al., 2022; Rogers et al., 2022; Wang et al., 2023).

Perhaps of more immediate concern regarding restored site permanence are short-term disturbances, such as stochastic storm impacts and anthropogenic pressures on restored coastal wetlands (Hanley et al., 2020; Newton et al., 2020). Minimizing the risk of such short-term perturbation will support permanence. If short-term perturbation risks can be minimized, sites with high sediment supply, large tide ranges, high rates of foundation species primary productivity, shallow elevation gradients, and harmonious upslope land use may both accumulate carbon rapidly and be resilient to future sea-level rise, retaining carbon in the long term (Cahoon et al., 2021; Osland et al., 2022; Saintilan et al., 2022). Overall, restoration may be most successful at achieving permanence when targeting areas where intertidal surfaces can readily adjust vertically and/or laterally through a combination of allochthonous and autochthonous processes, ensuring resilience through 2050. Projects that do submerge from relative sea-level rise after management-relevant timescales can still have important mitigation contributions over the next several decades.

### Local factors

Whether or not the fundamental requirements of additionality, feasibility and permanence are met by a restoration action is largely set by global and regional-scale factors. However, local-scale factors including restoration design and the identity/abundance of biota can enhance or detract from site-specific restoration effectiveness as NCS. Restoration design decisions can determine channel density and flow path, wetland elevation and inundation, and vegetation cover and community identity through planting or natural colonization approaches (Lester et al., 2020; Vanderklift et al., 2020; Valach et al., 2021), all of which can influence the net cooling benefit of restoration compared to initial conditions. Research exploring the impact that coastal wetland restoration design decisions have on restored site effectiveness as NCS could expand on what little is currently known about which designs maximize carbon sequestration. These studies may be especially informative if they focus explicitly on how design options influence additionality, feasibility and permanence.

Local interactions of environmental conditions with biota, including macrophytes, macrofauna and microbes, can influence restoration effectiveness as NCS as well. Vegetation influence on local-scale carbon dynamics is becoming better characterized (Jones et al., 2018; Mueller et al., 2020; Kennedy et al., 2022; Kong et al., 2022; Jeffrey et al., 2023), although current work is often less clear on the precise mechanisms of plant-mediation of carbon processes (but see Vroom et al., 2022). Foundation plant species often establish quickly after restoration, jump-starting wetland carbon uptake, but this is not universally true where foundation species are large and/or slow growing (Marbà et al., 1996; Ballanti et al., 2017). Less well known are macrofaunal influences on restoration effectiveness as NCS. A growing body of literature has emphasized the importance of crab bioturbation on carbon loss in tidal marshes and mangroves, for example, via changes in sediment permeability/exchange and microbial communities, among other mechanisms (Gutiérrez et al., 2006; Guimond et al., 2020; Xiao et al., 2021; Qin et al., 2024; Smith, 2024). Microbial processes, dependent upon the abundance and identity of microbial communities, vary at small spatial scales and are strongly influenced by tidal inundation and associated abiotic factors (e.g., water content, salinity, oxygen and nutrient availability; Cheung et al., 2018; Rinke et al., 2022). Following wetland restoration, changes can occur in fungal communities as well, as the ecosystem matures into a marine setting (Walker and Campbell, 2010; Dini-Andreote et al., 2016). Microbial communities may have a strong impact on restoration effectiveness as NCS by exerting a key influence on carbon cycling processes important for cooling benefits (e.g., methanogenesis and methane oxidation; Oremland and Polcin, 1982; Segarra et al., 2013; Capooci et al., 2024). Additional studies that explore how organism presence and abundance impact a site’s capacity to meet the fundamental NCS requirements within the range set by climate and geomorphology would be helpful.

## Future perspectives

Coastal wetland restoration will be most effective as NCS where additionality, feasibility and permanence are maximized. Verifying these requirements are met in an ecological context (Table 1) is more straightforward than the complex task of quantifying the magnitude of project-specific cooling benefits for carbon finance or accounting purposes. Issues with quantifying magnitudes of climate benefit are not addressed here, as we focus below on the issues impeding initial deliberation of whether restoration has a net climate benefit, the crucial point for restoration implementation.

### Coastal wetlands as cross-ecosystem linkages

One issue in understanding when and where coastal restoration actions are effective NCS is uncertainty in additionality for ecosystems that are interfaces and integrators of terrestrial and aquatic habitats. It is sometimes unclear if a restoration project meets the fundamental requirement of having a net cooling benefit when those cooling benefits can occur in habitats outside of project footprints. For example, connectivity between restored sites and surrounding landscapes can be an important driver of the carbon cycling benefits of restoration (Woo et al., 2022; Mazarrasa et al., 2023). Allochthonous material, in particular, can be buried at substantial rates upon initial restoration in salt marshes (Wollenberg et al., 2018; Mossman et al., 2022). The reduced water movement through seagrass meadow canopies (Peralta et al., 2008) not only facilitates high retention of autochthonous production, but also results in increased deposition of allochthonous carbon (Fonseca and Fisher, 1986; Hendriks et al., 2008), estimated to contribute to ~50% of the sediment organic C pool in these meadows on average (Kennedy et al., 2010). Tidal forests can also trap substantial amounts of allochthonous material (e.g., Noe et al., 2016). However, there remains uncertainty on whether allochthonous carbon removed from the atmosphere upstream or upslope and then buried in a coastal wetland should be considered part of the cooling benefit from the restoration action. Similarly, there remains uncertainty on if autochthonous carbon that is removed from the atmosphere in a coastal wetland restoration area and then exported laterally to the near-shore environment with potential long-term storage (especially as dissolved inorganic carbon or total alkalinity; Santos et al., 2019, 2021; Yau et al., 2022; Reithmaier et al., 2023) should be considered part of the cooling benefit. Ignoring these lateral connections can affect the estimated cooling benefit of a restoration action, potentially influencing if projects meet the fundamental requirement of additionality (Bogard et al., 2020; Schutte et al., 2020; Correa et al., 2022).

**Figure 2.**
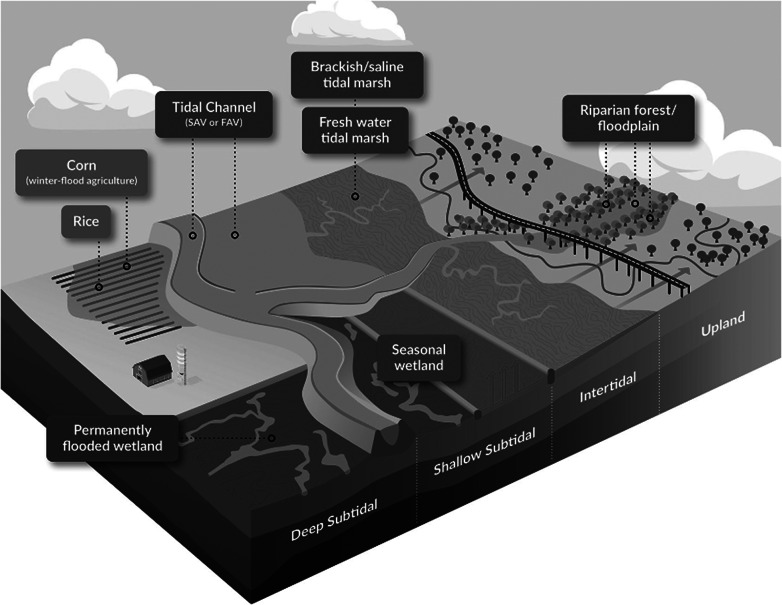
Land-use types of interest to carbon sequestration and/or GHG mitigation across the relative tidal elevation range in Suisun Bay and Delta lands. Corn indicates conventional row crops. Tidal channel refers to open-water aquatic habitats, whether deep or shallow (such as flooded islands) and which may be populated by submerged or floating aquatic vegetation (SAV and FAV). Permanently flooded wetland refers to wetlands impounded to reverse subsidence. Seasonal wetland refers to wetlands managed via freshwater flooding to benefit wildlife. *Credit: Illustrated by Vincent Pascual, California Office of State Publishing, adapted from SFEI*.

To address this issue, one approach is to take a landscape/systems view for determining if a specific management action will lead to a net cooling benefit (Figure 2), regardless of the spatial footprint that benefit occurs in (similar to the efforts underway for landscape-scale carbon accounting; Glass et al., 2024). In other words, tracking the response of a landscape (e.g., a watershed) to management actions, not the response of one habitat type to management actions. Lateral export of carbon that is buried (or emitted) outside of the restoration site should contribute to understanding a wetland’s cooling benefit compared to initial conditions, as long as the export would not have occurred without restoration action. In the case of greenhouse gas emissions, particular care must be taken to ensure appropriate baseline comparisons that consider surrounding land uses as potential sources contributing to wetland fluxes (e.g., N_2_O from prairie pothole wetlands; Tangen and Bansal, 2022), to avoid penalizing wetlands as the spot where allochthonous carbon enters the atmosphere even when the land management decisions driving carbon and nutrient export and mineralization are made upslope or upstream. For allochthonous carbon burial, if the accommodation space created by restoration allows the preservation of carbon that would have otherwise been mineralized, that leads to a cooling benefit even if the carbon was removed from the atmosphere offsite (Wollenberg et al., 2018). We acknowledge that taking a landscape approach to the cooling benefit of coastal wetland restoration may be difficult in practice, as mass balance approaches are most tractable at site-level scales. However, this approach may allow a more holistic understanding of additionality and cooling benefit from restoration actions in coastal wetlands, incorporating the true connectivity of these habitats as cross-ecosystem linkages.

### Coastal wetland migration

Another issue in understanding when and where coastal restoration actions are effective NCS is uncertainty in permanence for ecosystems that are dynamic on the landscape. As they cope with accelerating sea-level rise, coastal wetlands have the potential to migrate both upslope and upstream over management-relevant timeframes (Krauss et al., 2018; Gedan and Fernández‐Pascual, 2019; Osland et al., 2022; Wang et al., 2023). Restored wetlands may therefore move out of the original project footprint over time, making it difficult to estimate the longevity of cooling benefits from management actions. Restored coastal wetlands may submerge under relative sea-level rise rates above ~5–7 mm/year (Saintilan et al., 2022; Morris et al., 2023), converting to unvegetated flats (Haywood et al., 2020; Schoolmaster et al., 2022). The fate of wetland carbon cycling with such state change and concurrent potential erosion is not clear (Creamer et al., 2024), but cooling benefit losses may be at least partially offset by cooling benefit gains as upslope or upstream habitats are converted to new wetlands (Osland et al., 2022; Wang et al., 2023). In certain regions with large areas of accommodation space, in fact, relative sea-level rise may increase the total habitat of coastal wetlands (Schieder et al., 2018), although newly colonized habitat from upslope migration may initially have lower soil carbon accumulation rates than mature habitat (Sandi et al., 2021). The resilience of a restored wetland to submergence over management-relevant decadal scales, including the availability of accommodation space to migrate upslope or upstream, therefore becomes of prime importance.

To address this issue, a landscape/systems approach again may be helpful (Figure 2), but for permanence: not tying permanence of restoration cooling benefit to a specific spatial footprint, unless human constraints on wetland migration preclude movement of carbon benefits across the landscape. This approach incorporates the natural dynamism of coastal wetlands and the reality of complex landscapes. Dealing with disturbance and dynamism is not new for habitats used as NCS: forests also experience disturbances like wildfire that can release stored carbon (Hurteau et al., 2008). Unlike forests experiencing fire, it is possible that stored carbon from submerging wetlands can continue to be stored with lateral export to shallow ocean shelves (Santos et al., 2021). Existing remote sensing tools and analyses can help to identify priority areas where restoration could be resilient and extend the lifetime of restored wetlands undergoing relative sea-level rise impacts (e.g., Rogers et al., 2023b; Ganju et al., 2024). Taking a landscape approach to permanence provides an opportunity to move beyond single--habitat focused restoration, thus aggregating restoration action influence across the landscape (Thorslund et al., 2017). Framing permanence in a management-relevant context (i.e., the next few decades) addresses the concern that some wetlands will submerge in the future (especially by the end of the century; Saintilan et al., 2022); having enhanced uptake in the next few decades can buy time for more robust climate solutions even if additionality somewhat decreases with migration. Ultimately, a landscape approach prevents focusing on the storage of a particular molecule in a particular place, shifting perspective to the overall cooling benefit of a management action.

### Methane and management-relevant timeframes

Uncertainty in how to incorporate methane emissions is another issue preventing understanding of when and where coastal restoration actions are effective NCS. Methane is a potent but short-lived greenhouse gas (Neubauer and Megonigal, 2015), and becomes a crucial component of the cooling benefit of restoration actions given decadal management-relevant timescales. Regardless of methane emissions, wetlands commonly exhibit climate cooling impacts on geologic timescales (Frolking et al., 2006; Neubauer and Megonigal, 2015); on management-relevant timescales, however, methane emissions can significantly influence the efficacy of restoration activities as NCS (Schutte et al., 2020; Arias-Ortiz et al., 2021b). Microbial communities responding to environmental conditions control the balance between methane production (methanogenesis) and consumption (methanotrophy/oxidation) in soils, as they break down organic matter for energy (Oremland and Polcin, 1982; Segarra et al., 2013; Capooci et al., 2024; Hartman et al., 2024). There is broad agreement that salinity (often used as a proxy for sulfate concentrations) decreases methane emissions in coastal wetlands, even if mechanisms remain uncertain (Bartlett et al., 1987; Poffenbarger et al., 2011; Bridgham et al., 2013; Chuang et al., 2016; Rosentreter et al., 2021; Sanders-DeMott et al., 2022). Dominant plant communities can also control methane emissions, through plant-mediated gas fluxes. These fluxes can make up the dominant pathway of methane emissions to the atmosphere in coastal wetlands, as methane vents through herbaceous or woody tissues (Jeffrey et al., 2019; Mueller et al., 2020; Villa et al., 2020; Comer-Warner et al., 2022). Finally, it is becoming clear that lateral export of dissolved methane is a potentially important, but under-recognized, methane flux pathway (Santos et al., 2019; Schutte et al., 2020; Chen et al., 2022; Wang et al., 2022b). Especially in low-salinity conditions and/or with high productivity of wetland-adapted plants, methane can complicate understanding if restoration actions meet the basic requirement of NCS of having a net cooling benefit.

Incorporating methane emissions is crucial, but may be most helpful within the context of coastal restoration as NCS when focused on the cooling benefit of specific management actions. Methane emissions are not inherently bad, and productive low-salinity restored sites with high methane emissions may still provide large cooling benefits compared to pre-restoration conditions (Hemes et al., 2019; Günther et al., 2020; Arias-Ortiz et al., 2021b; Nyberg et al., 2022; Adame et al., 2024). Methane emissions are sometimes measured in restored coastal wetlands, but often pre-restoration baseline data or data from analog/alternative land use sites are lacking, preventing an understanding of the net change in methane emissions and overall cooling benefit attributable to restoration actions. Therefore, effective methane monitoring includes data collection at alternative land use sites and begins pre-restoration where possible. Additionally, coordinated synthesis activities can help in gathering, making available, and interpreting the rapidly accumulating greenhouse gas flux datasets from blue carbon ecosystems, especially for marshes and mangroves (Knox et al., 2019; Rosentreter et al., 2023; Arias-Ortiz et al., 2024). Seagrasses pose a particular challenge here, as they exchange dissolved inorganic carbon with the water column rather than carbon dioxide directly with the atmosphere. Coordinated synthesis of benthic, air–water, and lateral fluxes in seagrass ecosystems, including methane, can provide needed insight into their restoration benefit as NCS, as with lateral fluxes in blue carbon ecosystems more generally (Santos et al., 2021). Incorporating methane emissions over management-relevant timeframes (e.g., by using sustained-flux global warming potential for a 20-year time horizon; Neubauer, 2021) without forgetting that methane emissions do not inherently preclude effectiveness as NCS can help to move the field toward inclusion of all tidal wetlands that may provide climate mitigation benefits (Adame et al., 2024).

### Minimum data requirements

Information supporting the likelihood that a project will, at a minimum, lead to a cooling benefit is a prerequisite for taking restoration action as NCS, but it is unclear if the magnitude of cooling benefits also needs to be quantified before action takes place. Modeling is often used as a tool for guiding restoration decision-making, but some projects do not require modeling approaches to understand the binary outcome of whether or not an action will have a cooling benefit (e.g., Twomey et al., 2024). Coastal wetland restoration projects are already happening around the world without a modeled estimate of cooling benefit; this lack of carbon accounting does not influence whether or not a real climate benefit is occurring. In landscapes with multiple competing values, or where a high level of precision is needed, complex models are certainly required to understand if an action has a net benefit. In the cases where more complex modeling is required, several biogeochemical models designed for tidal wetlands enable the prediction of organic carbon accumulation, sediment accretion, and other carbon-related processes with changes in relative sea levels (Buffington et al., 2021; Morris et al., 2023; Vahsen et al., 2024). This particular scenario may be uncommon when considering all the locations where blue carbon restoration is likely to be successful globally. Where complex models are not required, there remains disagreement on the data necessary to understand project effectiveness as NCS. When plot-level data exist, an additional uncertainty is how best to use spatially explicit information to scale up to footprints relevant for projecting landscape-level response to restoration action (Duarte de Paula Costa et al., 2022; Matthes et al., 2014; Shahan et al., 2022). Regardless of the complexity of data required, long-term post-implementation monitoring allows evaluating actual restoration project responses and ensures projects are meeting expectations and targets over time (Wortley et al., 2013; Cadier et al., 2020; Lovelock et al., 2022c). A robust understanding of carbon cycling responses to restoration action is crucial for quantifying the total magnitude of cooling benefit, but where cooling benefit is not predicted to be large, it may be critical for understanding if a cooling benefit exists at all.

It may be useful to explicitly differentiate the minimum data requirements for coastal restoration as effective NCS (i.e., answering ‘does this management action accrue a climate benefit?’) from the data requirements for quantifying the magnitude of cooling benefits for carbon accounting purposes (i.e., answering ‘how much climate benefit does this management action accrue?’). There is a need for widely distributed, standardized minimum data that can be applied to address the former question (Table 1). Much of the minimum data needed is spatial in nature, as spatially explicit data are most useful to land managers and restoration practitioners for on-the-ground prioritization (Lovelock et al., 2022b; Rogers et al., 2023b). These spatial data include up-to-date maps of regional land use/land cover (Sleeter et al., 2018, 2022), land ownership (Lovelock and Brown, 2019), and topography (including human alterations that impede wetland migration; Osland et al., 2022; Rogers et al., 2023b). Vegetation types in particular are often mappable, and may be crucial to up-scale data on climate benefits using remote sensing observations (e.g., Kong et al., 2022). Other minimum data requirements are not explicitly spatial (but may still vary regionally), including carbon balance or emissions/removal factor estimates for land use/land cover classes (from direct measurements or model outputs; Hagger et al., 2022; Windham-Myers et al., 2023), estimates of restoration cost per area restored given the prevailing restoration culture and financial incentive (Taillardat et al., 2020; Hagger et al., 2022), and resilience to relative sea-level rise (Holmquist et al., 2021; Ganju et al., 2024). Further, communicating and collaborating with local communities to ensure stakeholder involvement and equitable distribution of restoration co-benefits is key in any project (Surgeon Rogers et al., 2019; Dencer-Brown et al., 2022; Lovelock et al., 2022c). Beyond these suggested standard data types, additional project-specific considerations that impact additionality, feasibility and permanence will arise. If minimum data requirements are unavailable in a region, that helps prioritize new data collection efforts. One way to fill gaps for areas without the minimum required data is to leverage areas with more intensive data. Using regional-scale data stratified by local-scale gradients like elevation, for example, can provide a path forward for estimating the value of restoration from a carbon perspective (Wang et al., 2022a; Lovelock et al., 2022b; Windham-Myers et al., 2023; Yando et al., 2023). Understanding the magnitude of cooling benefits for accounting purposes is a crucial, but distinct, second step in the process of addressing effectiveness of coastal wetland restoration as NCS. We posit that confounding these distinct questions can impede implementation of restoration projects that are likely to have a climate benefit.

## Conclusion

Here, we synthesize the fundamental requirements of additionality, feasibility and permanence to address the question, ‘when and where is coastal wetland restoration effective as a natural climate solution?’ Maximizing the values underpinning these three key factors can increase the effectiveness of restoration projects, for example, by targeting regions with large areas of degraded habitat that will net a substantial climate cooling benefit when restored (additionality); where socio-economic and governance factors are in place to support action (feasibility); and where there is high resilience to future relative sea-level rise (permanence). Recent work is leading the way for effective site-level prioritization (Rogers et al., 2019b; Moritsch et al., 2021; Duarte de Paula Costa et al., 2022; Rogers et al., 2023b). To move toward successful implementation at scale, we highlight paths forward on several issues impeding confidence in coastal wetland restoration as NCS. First, tracking the cooling benefit of specific management actions across the interconnected coastal landscape, not project-specific spatial footprints. Second, the importance of incorporating methane into restoration considerations, as effective NCS will function over management-relevant decadal timescales. Finally, the minimum data required to understand if an action has a climate benefit is likely more tractable than the data required to understand the separate issue of quantifying the magnitude of climate benefit. Ultimately, for maximal NCS effectiveness, energy and resources will be focused on prioritizing sites with high additionality, where restoration actions are feasible and where permanence is likely. We stress that within this framework, coastal wetland restoration provides immense benefits beyond mitigating climate change (Vegh et al., 2019; Pindilli, 2022; Hambäck et al., 2023). There are strong calls for ecosystem restoration over the next decade (United Nations Environment Programme (UNEP), 2021). Reducing uncertainty can help to ensure that coastal restoration actions deliver climate cooling benefits within the decadal timeframes necessary to function as one climate mitigation strategy among many.

## Data Availability

No data were used in the preparation of this manuscript.

## References

[r1] Abernethy S and Jackson RB (2022) Global temperature goals should determine the time horizons for greenhouse gas emission metrics. Environmental Research Letters 17(2), 024019. 10.1088/1748-9326/ac4940.

[r2] Adame MF, Kelleway J, Krauss KW, Lovelock CE, Adams JB, Trevathan-Tackett SM, Noe G, Jeffrey L, Ronan M, Zann M, Carnell PE, Iram N, Maher DT, Murdiyarso D, Sasmito S, Tran DB, Dargusch P, Kauffman JB and Brophy L (2024) All tidal wetlands are blue carbon ecosystems. BioScience 74(4), 253–268. 10.1093/biosci/biae007. 38720908 PMC11075650

[r3] Arias-Ortiz A, Masqué P, Glass L, Benson L, Kennedy H, Duarte CM, Garcia-Orellana J, Benitez-Nelson CR, Humphries MS, Ratefinjanahary I, Ravelonjatovo J and Lovelock CE (2021a) Losses of soil organic carbon with deforestation in mangroves of Madagascar. Ecosystems 24(1), 1–19. 10.1007/s10021-020-00500-z.

[r4] Arias-Ortiz A, Oikawa PY, Carlin J, Masqué P, Shahan J, Kanneg S, Paytan A and Baldocchi D (2021b) Tidal and nontidal marsh restoration: A trade‐off between carbon sequestration, methane emissions, and soil accretion. Journal of Geophysical Research: Biogeosciences 126, e2021JG006573.

[r195] Arias-Ortiz A, Wolfe J, Bridgham SD, Knox S, McNicol G, Needelman BA, … and Holmquist JR (2024) Methane fluxes in tidal marshes of the conterminous United States. Global Change Biology 30(9), e17462. 10.1111/gcb.17462. 39234688

[r5] Ballanti L, Byrd K, Woo I and Ellings C (2017) Remote sensing for wetland mapping and historical change detection at the Nisqually River Delta. Sustainability 9(11), 1919. 10.3390/su9111919.

[r6] Bartlett KB, Bartlett DS, Harriss RC and Sebacher DI(1987) Methane emissions along a salt marsh salinity gradient. Biogeochemistry 4(3), 183–202. 10.1007/BF02187365.

[r7] Bell-James J, Fitzsimons JA and Lovelock CE (2023) Land tenure, ownership and use as barriers to coastal wetland restoration projects in Australia: Recommendations and solutions. Environmental Management 72(1), 179–189. 10.1007/s00267-023-01817-w. 37010555 PMC10220139

[r8] Bogard MJ, Bergamaschi BA, Butman DE, Anderson F, Knox SH and Windham‐Myers L (2020) Hydrologic export is a major component of coastal wetland carbon budgets. Global Biogeochemical Cycles 34(8), e2019GB006430. 10.1029/2019GB006430.

[r9] Boyd R, Dalrymple R and Zaitlin BA (1992) Classification of clastic coastal depositional environments. Sedimentary Geology 80(3–4), 139–150. 10.1016/0037-0738(92)90037-R.

[r10] Breithaupt JL and Steinmuller HE (2022) Refining the global estimate of mangrove carbon burial rates using sedimentary and geomorphic settings. Geophysical Research Letters 49(18), e2022GL100177. 10.1029/2022GL100177.

[r11] Bridgham SD, Cadillo-Quiroz H, Keller JK and Zhuang Q (2013) Methane emissions from wetlands: biogeochemical, microbial, and modeling perspectives from local to global scales. Global Change Biology 19(5), 1325–1346. 10.1111/gcb.12131. 23505021

[r12] Bridgham SD, Megonigal JP, Keller JK, Bliss NB and Trettin C (2006) The carbon balance of North American wetlands. Wetlands 26(4), 889–916. 10.1672/0277-5212(2006)26[889:TCBONA]2.0.CO;2.

[r13] Broome SW, Seneca ED and Woodhouse WW (1988) Tidal salt marsh restoration. Aquatic Botany 32(1–2), 1–22. 10.1016/0304-3770(88)90085-X.

[r14] Buffington KJ, Janousek CN, Dugger BD, Callaway JC, Schile-Beers LM, Borgnis Sloane E and Thorne KM (2021) Incorporation of uncertainty to improve projections of tidal wetland elevation and carbon accumulation with sea-level rise. PLoS ONE 16(10), e0256707. 10.1371/journal.pone.0256707. 34669722 PMC8528310

[r15] Burden A, Garbutt A and Evans CD (2019) Effect of restoration on saltmarsh carbon accumulation in Eastern England. Biology Letters 15(1), 20180773. 10.1098/rsbl.2018.0773. 30907701 PMC6371915

[r16] Cadier C, Bayraktarov E, Piccolo R and Adame MF (2020) Indicators of coastal wetlands restoration success: A systematic review. Frontiers in Marine Science 7, 600220. 10.3389/fmars.2020.600220.

[r17] Cahoon DR, McKee KL and Morris JT (2021) How plants influence resilience of salt marsh and mangrove wetlands to sea-level rise. Estuaries and Coasts 44(4), 883–898. 10.1007/s12237-020-00834-w.

[r18] Campbell AD, Fatoyinbo L, Goldberg L and Lagomasino D (2022) Global hotspots of salt marsh change and carbon emissions. Nature 612(7941), 701–706. 10.1038/s41586-022-05355-z. 36450979 PMC9771810

[r19] Capooci M, Seyfferth AL, Tobias C, Wozniak AS, Hedgpeth A, Bowen M, Biddle JF, McFarlane KJ and Vargas R (2024) High methane concentrations in tidal salt marsh soils: Where does the methane go? Global Change Biology 30(1), e17050. 10.1111/gcb.17050. 38273533

[r20] Chambers LG, Steinmuller HE and Breithaupt JL (2019) Toward a mechanistic understanding of “peat collapse” and its potential contribution to coastal wetland loss. Ecology 100(7), e02720. 10.1002/ecy.2720. 30933312 PMC6850666

[r21] Chen X, Santos IR, Hu D, Zhan L, Zhang Y, Zhao Z, Hu S and Li L (2022) Pore‐water exchange flushes blue carbon from intertidal saltmarsh sediments into the sea. Limnology and Oceanography Letters 7(4), 312–320. 10.1002/lol2.10236.

[r22] Cheung MK, Wong CK, Chu KH and Kwan HS (2018) Community structure, dynamics and interactions of bacteria, archaea and fungi in subtropical coastal wetland sediments. Scientific Reports 8(1), 14397. 10.1038/s41598-018-32529-5. 30258074 PMC6158284

[r23] Chmura GL, Anisfeld SC, Cahoon DR and Lynch JC (2003) Global carbon sequestration in tidal, saline wetland soils. Global Biogeochemical Cycles 17(4), 1111. 10.1029/2002GB001917.

[r24] Chuang P-C, Young MB, Dale AW, Miller LG, Herrera-Silveira JA and Paytan A (2016) Methane and sulfate dynamics in sediments from mangrove-dominated tropical coastal lagoons, Yucatán, Mexico. Biogeosciences 13(10), 2981–3001. 10.5194/bg-13-2981-2016.

[r25] Claasens L, Adams JB, de Villiers NM, Wasserman J and Whitfield AK (2022) Restoration of South African estuaries: successes, failures and the way forward. African Journal of Aquatic Science 48(1), 1–18. 10.2989/16085914.2022.2115970.

[r26] Comer-Warner SA, Ullah S, Ampuero Reyes W, Krause S and Chmura GL (2022) Spartina alterniflora has the highest methane emissions in a St. Lawrence estuary salt marsh. Environmental Research: Ecology 1(1), 011003. 10.1088/2752-664X/ac706a.

[r27] Cormier N, Krauss KW, Demopoulos A, Jessen BJ, McClain-Counts JP, From AS and Flynn LL (2022) Potential for carbon and nitrogen sequestration by restoring tidal connectivity and enhancing soil surface elevations in denuded and degraded South Florida mangrove ecosystems. In Krauss KW, Zhu Z, Stagg CL (eds.), Wetland Carbon and Environmental Management. Hoboken, NJ: American Geophysical Union and John Wiley and Sons, Inc., pp. 143–158.

[r28] Correa RE, Xiao K, Conrad SR, Wadnerkar PD, Wilson AM, Sanders CJ and Santos IR (2022) Groundwater carbon exports exceed sediment carbon burial in a salt marsh. Estuaries and Coasts 45(6), 1545–1561. 10.1007/s12237-021-01021-1.

[r29] Creamer CA, Waldrop MP, Stagg CL, Manies KL, Baustian MM, Laurenzano C, Aw TG, Haw M, Merino SL, Schoolmaster DR, Sevilgen S, Villani RK and Ward EJ (2024) Vegetation loss following vertical drowning of Mississippi River deltaic wetlands leads to faster microbial decomposition and decreases in soil carbon. Journal of Geophysical Research: Biogeosciences 129(4), e2023JG007832. 10.1029/2023JG007832.

[r30] Dencer-Brown AM, Shilland R, Friess D, Herr D, Benson L, Berry NJ, Cifuentes-Jara M, Colas P, Damayanti E, García EL, Gavaldão M, Grimsditch G, Hejnowicz AP, Howard J, Islam ST, Kennedy H, Kivugo RR, Lang’at JKS, Lovelock C, Malleson R, Macreadie PI, Andrade-Medina R, Mohamed A, Pidgeon E, Ramos J, Rosette M, Salim MM, Schoof E, Talukder B, Thomas T, Vanderklift MA and Huxham M (2022) Integrating blue: How do we make nationally determined contributions work for both blue carbon and local coastal communities? Ambio 51(9), 1978–1993. 10.1007/s13280-022-01723-1. 35503201 PMC9063623

[r31] Diffenbaugh NS and Barnes EA (2023) Data-driven predictions of the time remaining until critical global warming thresholds are reached. Proceedings of the National Academy of Sciences 120(6), e2207183120. 10.1073/pnas.2207183120. PMC9963891 36716375

[r32] Dini-Andreote F, Pylro VS, Baldrian P, van Elsas JD and Salles JF (2016) Ecological succession reveals potential signatures of marine–terrestrial transition in salt marsh fungal communities. The ISME Journal 10(8), 1984–1997. 10.1038/ismej.2015.254. 26824176 PMC5029165

[r33] Dittmann S, Mosley L, Beaumont K, Bestland E, Guan H, Sandhu H, Clanahan M, Baring R, Quinn J, Sutton P, Thomson SM, Shepherd G, Whalen M, Marschner P and Townsend M (2019) From salt to C; carbon sequestration through ecological restoration at the Dry Creek Salt Field. Goyder Institute for Water Research, Technical Report Series, n. 19/28, 113 p.

[r34] Drexler JZ, Fontaine CS and Deverel SJ (2009) The legacy of wetland drainage on the remaining peat in the Sacramento — San Joaquin Delta, California, USA. Wetlands 29(1), 372–386. 10.1672/08-97.1.

[r35] Duarte de Paula Costa M, Lovelock CE, Waltham NJ, Moritsch MM, Butler D, Power T, Thomas E and Macreadie PI (2022) Modelling blue carbon farming opportunities at different spatial scales. Journal of Environmental Management 301, 113813. 10.1016/j.jenvman.2021.113813. 34607133

[r36] Eagle MJ, Kroeger KD, Spivak AC, Wang F, Tang J, Abdul-Aziz OI, Ishtiaq KS, O’Keefe Suttles J and Mann AG (2022) Soil carbon consequences of historic hydrologic impairment and recent restoration in coastal wetlands. Science of the Total Environment 848, 157682. 10.1016/j.scitotenv.2022.157682. 35917962

[r37] Ellis PW, Page AM, Wood S, Fargione J, Masuda YJ, Carrasco Denney V, Moore C, Kroeger T, Griscom B, Sanderman J, Atleo T, Cortez R, Leavitt S and Cook-Patton SC (2024) The principles of natural climate solutions. Nature Communications 15(1), 547. 10.1038/s41467-023-44425-2. PMC10805724 38263156

[r38] Fard E, Brown LN, Ambrose RF, Whitcraft C, Thorne KM, Kemnitz NJ, Hammond DE and MacDonald GM (2024) Increasing salt marsh elevation using sediment augmentation: Critical insights from surface sediments and sediment cores. Environmental Management 73(3), 614–633. 10.1007/s00267-023-01897-8. 37910218 PMC10884093

[r39] Fargione JE, Bassett S, Boucher T, Bridgham SD, Conant RT, Cook-Patton SC, Ellis PW, Falcucci A, Fourqurean JW, Gopalakrishna T, Gu H, Henderson B, Hurteau MD, Kroeger KD, Kroeger T, Lark TJ, Leavitt SM, Lomax G, McDonald RI, Megonigal JP, Miteva DA, Richardson CJ, Sanderman J, Shoch D, Spawn SA, Veldman JW, Williams CA, Woodbury PB, Zganjar C, Baranski M, Elias P, Houghton RA, Landis E, McGlynn E, Schlesinger WH, Siikamaki JV, Sutton-Grier AE and Griscom BW (2018) Natural climate solutions for the United States. Science Advances 4(11), eaat1869. 10.1126/sciadv.aat1869. 30443593 PMC6235523

[r40] Fonseca M and Fisher J (1986) A comparison of canopy friction and sediment movement between four species of seagrass with reference to their ecology and restoration. Marine Ecology Progress Series 29, 15–22. 10.3354/meps029015.

[r41] Friess DA, Howard J, Huxham M, Macreadie PI and Ross F (2022) Capitalizing on the global financial interest in blue carbon. PLOS Climate 1(8), e0000061. 10.1371/journal.pclm.0000061.

[r42] Friess DA, Rogers K, Lovelock CE, Krauss KW, Hamilton SE, Lee SY, Lucas R, Primavera J, Rajkaran A and Shi S (2019) The state of the world’s mangrove forests: Past, present, and future. Annual Review of Environment and Resources 44(1), 89–115. 10.1146/annurev-environ-101718-033302.

[r43] Frolking S, Roulet N and Fuglestvedt J (2006) How northern peatlands influence the earth’s radiative budget: Sustained methane emission versus sustained carbon sequestration. Journal of Geophysical Research: Biogeosciences 111(G1), 2005JG000091. 10.1029/2005JG000091.

[r44] Gamble C, Debney A, Glover A, Bertelli C, Green B, Hendy I, Lilley R, Nuuttila H, Potouroglou M, Ragazzola F, Unsworth R and Preston J (eds) (2021) Seagrass Restoration Handbook: UK & Ireland. London: Zoological Society of London.

[r45] Ganju NK, Ackerman KV and Defne Z (2024) Using geospatial analysis to guide marsh restoration in Chesapeake Bay and beyond. Estuaries and Coasts 47(1), 1–17. 10.1007/s12237-023-01275-x.

[r46] Gedan KB and Fernández‐Pascual E (2019) Salt marsh migration into salinized agricultural fields: A novel assembly of plant communities. Journal of Vegetation Science 30(5), 1007–1016. 10.1111/jvs.12774.

[r47] Glass L, Emmer I, Howard J and Tonneijck F (2024) Landscape GHG Accounting Guidance: How to Develop Landscape-Scale Carbon Projects. Wageningen, The Netherlands: Wetlands International, 44 p.

[r48] Gorham C, Lavery PS, Kelleway JJ, Masque P and Serrano O (2021) Heterogeneous tidal marsh soil organic carbon accumulation among and within temperate estuaries in Australia. Science of the Total Environment 787, 147482. 10.1016/j.scitotenv.2021.147482.

[r49] Graf A, Wohlfahrt G, Aranda-Barranco S, Arriga N, Brümmer C, Ceschia E, Ciais P, Desai AR, Di Lonardo S, Gharun M, Grünwald T, Hörtnagl L, Kasak K, Klosterhalfen A, Knohl A, Kowalska N, Leuchner M, Lindroth A, Mauder M, Migliavacca M, Morel AC, Pfennig A, Poorter H, Terán CP, Reitz O, Rebmann C, Sanchez-Azofeifa A, Schmidt M, Šigut L, Tomelleri E, Yu K, Varlagin A and Vereecken H (2023) Joint optimization of land carbon uptake and albedo can help achieve moderate instantaneous and long-term cooling effects. Communications Earth & Environment 4(1), 298. 10.1038/s43247-023-00958-4. 38665193 PMC11041785

[r50] Guimond JA, Seyfferth AL, Moffett KB and Michael HA (2020) A physical-biogeochemical mechanism for negative feedback between marsh crabs and carbon storage. Environmental Research Letters 15(3), 034024. 10.1088/1748-9326/ab60e2.

[r51] Günther A, Barthelmes A, Huth V, Joosten H, Jurasinski G, Koebsch F and Couwenberg J (2020) Prompt rewetting of drained peatlands reduces climate warming despite methane emissions. Nature Communications 11(1), 1644. 10.1038/s41467-020-15499-z. PMC7118086 32242055

[r52] Gutiérrez JL, Jones CG, Groffman PM, Findlay SEG, Iribarne OO, Ribeiro PD and Bruschetti CM (2006) The contribution of crab burrow excavation to carbon availability in surficial salt-marsh sediments. Ecosystems 9(4), 647–658. 10.1007/s10021-006-0135-9.

[r53] Hagger V, Waltham NJ and Lovelock CE (2022) Opportunities for coastal wetland restoration for blue carbon with co-benefits for biodiversity, coastal fisheries, and water quality. Ecosystem Services 55, 101423. 10.1016/j.ecoser.2022.101423.

[r54] Hambäck PA, Dawson L, Geranmayeh P, Jarsjö J, Kačergytė I, Peacock M, Collentine D, Destouni G, Futter M, Hugelius G, Hedman S, Jonsson S, Klatt BK, Lindström A, Nilsson JE, Pärt T, Schneider LD, Strand JA, Urrutia-Cordero P, Åhlén D, Åhlén I and Blicharska M (2023) Tradeoffs and synergies in wetland multifunctionality: A scaling issue. Science of the Total Environment 862, 160746. 10.1016/j.scitotenv.2022.160746. 36513236

[r55] Hanley ME, Bouma TJ and Mossman HL (2020) The gathering storm: optimizing management of coastal ecosystems in the face of a climate-driven threat. Annals of Botany 125(2), 197–212. 10.1093/aob/mcz204. 31837218 PMC6996050

[r56] Hartman WH, Bueno de Mesquita CP, Theroux SM, Morgan-Lang C, Baldocchi DD and Tringe SG (2024) Multiple microbial guilds mediate soil methane cycling along a wetland salinity gradient. MSystems 9(1), e00936-23. 10.1128/msystems.00936-23. PMC10804969 38170982

[r57] Haywood BJ, Hayes MP, White JR and Cook RL (2020) Potential fate of wetland soil carbon in a deltaic coastal wetland subjected to high relative sea level rise. Science of the Total Environment 711, 135185. 10.1016/j.scitotenv.2019.135185. 31831247

[r58] Hemes KS, Chamberlain SD, Eichelmann E, Anthony T, Valach A, Kasak K, Szutu D, Verfaillie J, Silver WL and Baldocchi DD (2019) Assessing the carbon and climate benefit of restoring degraded agricultural peat soils to managed wetlands. Agricultural and Forest Meteorology 268, 202–214. 10.1016/j.agrformet.2019.01.017.

[r59] Hendriks I, Sintes T, Bouma T and Duarte C (2008) Experimental assessment and modeling evaluation of the effects of the seagrass *Posidonia oceanica* on flow and particle trapping. Marine Ecology Progress Series 356, 163–173. 10.3354/meps07316.

[r60] Herr D, Vegh T and Von Unger M (2019) State of international policy for blue carbon actions. In Windham-Myers L, Crooks S, Troxler T (eds.), A Blue Carbon Primer: The State of Coastal Wetland Carbon Science, Practice and Policy. Boca Raton, FL: CRC Press, Taylor & Francis Group, pp. 199–215.

[r61] Holmquist JR, Brown LN and MacDonald GM (2021) Localized scenarios and latitudinal patterns of vertical and lateral resilience of tidal marshes to sea‐level rise in the contiguous United States. Earth’s Future 9(6), e2020EF001804. 10.1029/2020EF001804.

[r62] Holmquist JR, Eagle M, Molinari RL, Nick SK, Stachowicz LC and Kroeger KD (2023) Mapping methane reduction potential of tidal wetland restoration in the United States. Communications Earth & Environment 4(1), 353. 10.1038/s43247-023-00988-y.

[r63] Hudson R, Kenworthy J and Best M (eds) (2021) Saltmarsh Restoration Handbook: UK and Ireland. Bristol: Environment Agency.

[r64] Hupp CR, Kroes DE, Noe GB, Schenk ER and Day RH (2019) Sediment trapping and carbon sequestration in floodplains of the lower Atchafalaya Basin, LA: Allochthonous versus autochthonous carbon sources. Journal of Geophysical Research: Biogeosciences 124(3), 663–677. 10.1029/2018JG004533.

[r194] Hurteau, MD, Koch GW and Hungate BA (2008) Carbon protection and fire risk reduction: toward a full accounting of forest carbon offsets. Frontiers in Ecology and the Environment 6(9), 493–498.

[r65] Intergovernmental Panel on Climate Change (IPCC) (2021) Climate Change 2021: The Physical Science Basis. Contribution of Working Group I to the Sixth Assessment Report of the Intergovernmental Panel on Climate Change. Cambridge, UK and New York, NY: Cambridge University Press.

[r66] Intergovernmental Panel on Climate Change (IPCC) (2022) Global Warming of 1.5C: An IPCC Special Report. Cambridge, UK and New York, NY: Cambridge University Press.

[r67] Janousek CN, Bailey SJ and Brophy LS (2020) Early ecosystem development varies with elevation and pre-restoration land use/land cover in a Pacific northwest tidal wetland restoration project. Estuaries and Coasts 44(4), 13–29. 10.1007/s12237-020-00782-5.

[r68] Jeffrey LC, Maher DT, Johnston SG, Kelaher BP, Steven A and Tait DR (2019) Wetland methane emissions dominated by plant‐mediated fluxes: Contrasting emissions pathways and seasons within a shallow freshwater subtropical wetland. Limnology and Oceanography 64, 1895–1912.

[r69] Jeffrey LC, Moras C, Tait DR, Johnston SG, Call M, Sippo JZ, Jeffrey N, Laicher-Edwards D and Maher DT (2023, September 13) Large methane emissions from tree stems complicate the wetland methane budget. Journal of Geophysical Research: Biogeosciences 128(12), e2023JG007679. 10.22541/essoar.169462059.96647067/v1.

[r70] Jia M, Wang Z, Mao D, Ren C, Song K, Zhao C, Wang C, Xiao X and Wang Y (2023) Mapping global distribution of mangrove forests at 10-m resolution. Science Bulletin 68(12), 1306–1316. 10.1016/j.scib.2023.05.004. 37217429

[r71] Jones SF, Stagg CL, Krauss KW and Hester MW (2018) Flooding alters plant-mediated carbon cycling independently of elevated atmospheric CO _2_ concentrations. Journal of Geophysical Research: Biogeosciences 123(6), 1976–1987. 10.1029/2017JG004369.

[r72] Kennedy H, Beggins J, Duarte CM, Fourqurean JW, Holmer M, Marbà N and Middelburg JJ (2010) Seagrass sediments as a global carbon sink: Isotopic constraints. Global Biogeochemical Cycles 24(4), 2010GB003848. 10.1029/2010GB003848.

[r73] Kennedy H, Pagès JF, Lagomasino D, Arias‐Ortiz A, Colarusso P, Fourqurean JW, Githaiga MN, Howard JL, Krause‐Jensen D, Kuwae T, Lavery PS, Macreadie PI, Marbà N, Masqué P, Mazarrasa I, Miyajima T, Serrano O and Duarte CM (2022) Species traits and geomorphic setting as drivers of global soil carbon stocks in seagrass meadows. Global Biogeochemical Cycles 36(10), e2022GB007481. 10.1029/2022GB007481.

[r74] Knox SH, Bansal S, McNicol G, Schafer K, Sturtevant C, Ueyama M, Valach AC, Baldocchi D, Delwiche K, Desai AR, Euskirchen E, Liu J, Lohila A, Malhotra A, Melling L, Riley W, Runkle BRK, Turner J, Vargas R, Zhu Q, Alto T, Fluet‐Chouinard E, Goeckede M, Melton JR, Sonnentag O, Vesala T, Ward E, Zhang Z, Feron S, Ouyang Z, Alekseychik P, Aurela M, Bohrer G, Campbell DI, Chen J, Chu H, Dalmagro HJ, Goodrich JP, Gottschalk P, Hirano T, Iwata H, Jurasinski G, Kang M, Koebsch F, Mammarella I, Nilsson MB, Ono K, Peichl M, Peltola O, Ryu Y, Sachs T, Sakabe A, Sparks JP, Tuittila E, Vourlitis GL, Wong GX, Windham‐Myers L, Poulter B and Jackson RB (2021) Identifying dominant environmental predictors of freshwater wetland methane fluxes across diurnal to seasonal time scales. Global Change Biology 27(15), 3582–3604. 10.1111/gcb.15661. 33914985

[r75] Knox SH, Jackson RB, Poulter B, McNicol G, Fluet-Chouinard E, Zhang Z, Hugelius G, Bousquet P, Canadell JG, Saunois M, Papale D, Chu H, Keenan TF, Baldocchi D, Torn MS, Mammarella I, Trotta C, Aurela M, Bohrer G, Campbell DI, Cescatti A, Chamberlain S, Chen J, Chen W, Dengel S, Desai AR, Euskirchen E, Friborg T, Gasbarra D, Goded I, Goeckede M, Heimann M, Helbig M, Hirano T, Hollinger DY, Iwata H, Kang M, Klatt J, Krauss KW, Kutzbach L, Lohila A, Mitra B, Morin TH, Nilsson MB, Niu S, Noormets A, Oechel WC, Peichl M, Peltola O, Reba ML, Richardson AD, Runkle BRK, Ryu Y, Sachs T, Schafer KVR, Schmid HP, Shurpali N, Sonnentag O, Tang ACI, Ueyama M, Vargas R, Vesala T, Ward EJ, Windham-Myers L, Wolhfahrt G and Zona D (2019) FLUXNET-CH4 synthesis activity: Objectives, observations, and future directions. Bulletin of the American Meteorological Society 100, 2607–2632. 10.1175/BAMS-D-18-0268.1.

[r76] Kong J, Ryu Y, Liu J, Dechant B, Rey-Sanchez C, Shortt R, Szutu D, Verfaillie J, Houborg R and Baldocchi DD (2022) Matching high resolution satellite data and flux tower footprints improves their agreement in photosynthesis estimates. Agricultural and Forest Meteorology 316, 108878. 10.1016/j.agrformet.2022.108878.

[r77] Krauss KW, Cormier N, Osland MJ, Kirwan ML, Stagg CL, Nestlerode JA, Russell MJ, From AS, Spivak AC, Dantin DD, Harvey JE and Almario AE (2017) Created mangrove wetlands store belowground carbon and surface elevation change enables them to adjust to sea-level rise. Scientific Reports 7(1), 1030. 10.1038/s41598-017-01224-2. 28432292 PMC5430729

[r78] Krauss KW, Lovelock CE, Chen L, Berger U, Ball MC, Reef R, Peters R, Bowen H, Vovides AG, Ward EJ, Wimmler M-C, Carr J, Bunting P and Duberstein JA (2022a) Mangroves provide blue carbon ecological value at a low freshwater cost. Scientific Reports 12(1), 17636. 10.1038/s41598-022-21514-8. 36271232 PMC9586979

[r79] Krauss KW, Noe GB, Duberstein JA, Conner WH, Stagg CL, Cormier N, Jones MC, Bernhardt CE, Graeme Lockaby B, From AS, Doyle TW, Day RH, Ensign SH, Pierfelice KN, Hupp CR, Chow AT and Whitbeck JL (2018) The role of the upper tidal estuary in wetland blue carbon storage and flux. Global Biogeochemical Cycles 32(5), 817–839. 10.1029/2018GB005897.

[r80] Krauss KW, Zhu Z and Stagg CL (eds) (2022b) Wetland Carbon and Environmental Management. Hoboken, NJ: American Geophysical Union and John Wiley & Sons, Inc.

[r81] Kroeger KD, Crooks S, Moseman-Valtierra S and Tang J (2017) Restoring tides to reduce methane emissions in impounded wetlands: A new and potent blue carbon climate change intervention. Scientific Reports 7(1), 11914. 10.1038/s41598-017-12138-4. 28931842 PMC5607314

[r82] Lester SE, Dubel AK, Hernán G, McHenry J and Rassweiler A (2020) Spatial planning principles for marine ecosystem restoration. Frontiers in Marine Science 7, 328. 10.3389/fmars.2020.00328.

[r83] Lewis RR, Milbrandt EC, Brown B, Krauss KW, Rovai AS, Beever JW and Flynn LL (2016) Stress in mangrove forests: Early detection and preemptive rehabilitation are essential for future successful worldwide mangrove forest management. Marine Pollution Bulletin 109(2), 764–771. 10.1016/j.marpolbul.2016.03.006. 26971817

[r84] Liu Z, Fagherazzi S and Cui B (2021) Success of coastal wetlands restoration is driven by sediment availability. Communications Earth & Environment 2(1), 44. 10.1038/s43247-021-00117-7.

[r85] Lovelock CE, Adame MF, Bradley J, Dittmann S, Hagger V, Hickey SM, Hutley LB, Jones A, Kelleway JJ, Lavery PS, Macreadie PI, Maher DT, McGinley S, McGlashan A, Perry S, Mosley L, Rogers K and Sippo JZ (2022a) An Australian blue carbon method to estimate climate change mitigation benefits of coastal wetland restoration. Restoration Ecology 31, e13739. 10.1111/rec.13739.

[r86] Lovelock CE, Adame MF, Butler DW, Kelleway JJ, Dittmann S, Fest B, King KJ, Macreadie PI, Mitchell K, Newnham M, Ola A, Owers CJ and Welti N (2022b) Modeled approaches to estimating blue carbon accumulation with mangrove restoration to support a blue carbon accounting method for Australia. Limnology and Oceanography 67(S2), S50–S60. 10.1002/lno.12014.

[r87] Lovelock CE, Barbier E and Duarte CM (2022c) Tackling the mangrove restoration challenge. PLOS Biology 20(10), e3001836. 10.1371/journal.pbio.3001836. 36251664 PMC9576054

[r88] Lovelock CE and Brown BM (2019) Land tenure considerations are key to successful mangrove restoration. Nature Ecology & Evolution 3(8), 1135–1135. 10.1038/s41559-019-0942-y. 31285575

[r89] Lovelock CE and Duarte CM (2019) Dimensions of blue carbon and emerging perspectives. Biology Letters 15(3), 20180781. 10.1098/rsbl.2018.0781. 30836882 PMC6451379

[r90] Macreadie PI, Costa MDP, Atwood TB, Friess DA, Kelleway JJ, Kennedy H, Lovelock CE, Serrano O and Duarte CM (2021) Blue carbon as a natural climate solution. Nature Reviews Earth & Environment 2(12), 826–839. 10.1038/s43017-021-00224-1.

[r91] Macreadie PI, Nielsen DA, Kelleway JJ, Atwood TB, Seymour JR, Petrou K, Connolly RM, Thomson AC, Trevathan‐Tackett SM and Ralph PJ (2017) Can we manage coastal ecosystems to sequester more blue carbon? Frontiers in Ecology and the Environment 15(4), 206–213. 10.1002/fee.1484.

[r92] Macreadie PI, Robertson AI, Spinks B, Adams MP, Atchison JM, Bell-James J, Bryan BA, Chu L, Filbee-Dexter K, Drake L, Duarte CM, Friess DA, Gonzalez F, Grafton RQ, Helmstedt KJ, Kaebernick M, Kelleway J, Kendrick GA, Kennedy H, Lovelock CE, Megonigal JP, Maher DT, Pidgeon E, Rogers AA, Sturgiss R, Trevathan-Tackett SM, Wartman M, Wilson KA and Rogers K (2022) Operationalizing marketable blue carbon. One Earth 5(5), 485–492. 10.1016/j.oneear.2022.04.005.

[r93] Manning WD, Scott CR and Leegwater E (eds) (2021) Restoring Estuarine and Coastal Habitats with Dredged Sediment: A Handbook. Bristol: Environment Agency.

[r94] Marbà N, Arias‐Ortiz A, Masqué P, Kendrick GA, Mazarrasa I, Bastyan GR, Garcia‐Orellana J and Duarte CM (2015) Impact of seagrass loss and subsequent revegetation on carbon sequestration and stocks. Journal of Ecology 103(2), 296–302. 10.1111/1365-2745.12370.

[r95] Marbà N, Duarte CM, Cebrian J, Gallegos ME, Olesen B and Sand-Jensen K (1996) Growth and population dynamics of *Posidonia oceanica* on the Spanish Mediterranean coast: elucidating seagrass decline. Marine Ecology Progress Series 137, 203–213.

[r96] Masselink G, Hanley ME, Halwyn AC, Blake W, Kingston K, Newton T and Williams M (2017) Evaluation of salt marsh restoration by means of self-regulating tidal gate – Avon estuary, South Devon, UK. Ecological Engineering 106, 174–190. 10.1016/j.ecoleng.2017.05.038.

[r97] Matthes JH, Sturtevant C, Verfaillie J, Knox S and Baldocchi D (2014) Parsing the variability in CH _4_ flux at a spatially heterogeneous wetland: Integrating multiple eddy covariance towers with high-resolution flux footprint analysis. Journal of Geophysical Research: Biogeosciences 119(7), 1322–1339. 10.1002/2014JG002642.

[r98] Mazarrasa I, Neto JM, Bouma TJ, Grandjean T, Garcia-Orellana J, Masqué P, Recio M, Serrano Ó, Puente A and Juanes JA (2023) Drivers of variability in blue carbon stocks and burial rates across European estuarine habitats. Science of the Total Environment 886, 163957. 10.1016/j.scitotenv.2023.163957. 37164078

[r99] McKenzie LJ, Nordlund LM, Jones BL, Cullen-Unsworth LC, Roelfsema C and Unsworth RKF (2020) The global distribution of seagrass meadows. Environmental Research Letters 15(7), 074041. 10.1088/1748-9326/ab7d06.

[r100] Mcleod E, Chmura GL, Bouillon S, Salm R, Björk M, Duarte CM, Lovelock CE, Schlesinger WH and Silliman BR (2011) A blueprint for blue carbon: toward an improved understanding of the role of vegetated coastal habitats in sequestering CO_2_. Frontiers in Ecology and the Environment 9(10), 552–560. 10.1890/110004.

[r101] Miller RL and Fujii R (2011) Re-establishing marshes can turn a current carbon source into a carbon sink in the Sacramento-San Joaquin Delta of California, USA. In Contreras DA (ed.), River Deltas: Types, Structures and Ecology. Hauppauge, NY: Nova Science Publishers, Inc., pp. 1–34.

[r102] Montague CL, Zale AV and Percival HF (1987) Ecological effects of coastal marsh impoundments: A review. Environmental Management 11, 743–756. 10.1007/BF01867242

[r103] Moritsch MM, Young M, Carnell P, Macreadie PI, Lovelock C, Nicholson E, Raimondi PT, Wedding LM and Ierodiaconou D (2021) Estimating blue carbon sequestration under coastal management scenarios. Science of the Total Environment 777, 145962. 10.1016/j.scitotenv.2021.145962. 33684760

[r104] Morris JT, Langley JA, Vervaeke WC, Dix N, Feller IC, Marcum P and Chapman SK (2023) Mangrove trees outperform saltmarsh grasses in building elevation but collapse rapidly under high rates of sea‐level rise. Earth’s Future 11(4), e2022EF003202. 10.1029/2022EF003202.

[r105] Mossman HL, Pontee N, Born K, Hill C, Lawrence PJ, Rae S, Scott J, Serato B, Sparkes RB, Sullivan MJP and Dunk RM (2022) Rapid carbon accumulation at a saltmarsh restored by managed realignment exceeded carbon emitted in direct site construction. PLOS ONE 17(11), e0259033. 10.1371/journal.pone.0259033. 36449465 PMC9710768

[r106] Mueller P, Mozdzer TJ, Langley JA, Aoki LR, Noyce GL and Megonigal JP (2020) Plant species determine tidal wetland methane response to sea level rise. Nature Communications 11(1), 5154. 10.1038/s41467-020-18763-4. PMC7560622 33056993

[r107] Neubauer SC (2021) Global warming potential is not an ecosystem property. Ecosystems 24(8), 2079–2089. 10.1007/s10021-021-00631-x.

[r108] Neubauer SC and Megonigal JP (2015) Moving beyond global warming potentials to quantify the climatic role of ecosystems. Ecosystems 18(6), 1000–1013. 10.1007/s10021-015-9879-4.

[r109] Newton A, Icely J, Cristina S, Perillo GME, Turner RE, Ashan D, Cragg S, Luo Y, Tu C, Li Y, Zhang H, Ramesh R, Forbes DL, Solidoro C, Béjaoui B, Gao S, Pastres R, Kelsey H, Taillie D, Nhan N, Brito AC, de Lima R and Kuenzer C (2020) Anthropogenic, direct pressures on coastal wetlands. Frontiers in Ecology and Evolution 8, 144. 10.3389/fevo.2020.00144.

[r110] Noe GB, Hupp CR, Bernhardt CE and Krauss KW (2016) Contemporary deposition and long-term accumulation of sediment and nutrients by tidal freshwater forested wetlands impacted by sea level rise. Estuaries and Coasts 39(4), 1006–1019. 10.1007/s12237-016-0066-4.

[r111] Novick KA, Keenan TF, Anderegg WRL, Normile CP, Runkle BRK, Oldfield EE, Shrestha G, Baldocchi DD, Evans MEK, Randerson JT, Sanderman J, Torn MS, Trugman AT and Williams CA (2024) We need a solid scientific basis for nature-based climate solutions in the United States. Proceedings of the National Academy of Sciences 121(14), e2318505121. 10.1073/pnas.2318505121. PMC10998553 38536749

[r112] Nyberg M, Black TA, Ketler R, Lee S ‐C., Johnson M, Merkens M, Nugent KA and Knox SH (2022) Impacts of active versus passive re‐wetting on the carbon balance of a previously drained bog. Journal of Geophysical Research: Biogeosciences 127(9), e2022JG006881. 10.1029/2022JG006881.

[r113] O’Brien EL and Zedler JB (2006) Accelerating the restoration of vegetation in a southern California salt marsh. Wetlands Ecology and Management 14(3), 269–286. 10.1007/s11273-005-1480-8.

[r114] Oremland RS and Polcin S (1982) Methanogenesis and sulfate reduction: Competitive and noncompetitive substrates in estuarine sediments. Applied and Environmental Microbiology 44(6), 1270–1276. 10.1128/aem.44.6.1270-1276.1982. 16346144 PMC242184

[r115] Orth R, Moore K, Marion S, Wilcox D and Parrish D (2012) Seed addition facilitates eelgrass recovery in a coastal bay system. Marine Ecology Progress Series 448, 177–195. 10.3354/meps09522.

[r116] Osland MJ, Chivoiu B, Enwright NM, Thorne KM, Guntenspergen GR, Grace JB, Dale LL, Brooks W, Herold N, Day JW, Sklar FH and Swarzenzki CM (2022) Migration and transformation of coastal wetlands in response to rising seas. Science Advances 8(26), eabo5174. 10.1126/sciadv.abo5174. 35767619 PMC9242587

[r117] Osland MJ, Feher LC, Spivak AC, Nestlerode JA, Almario AE, Cormier N, From AS, Krauss KW, Russell MJ, Alvarez F, Dantin DD, Harvey JE and Stagg CL (2020) Rapid peat development beneath created, maturing mangrove forests: Ecosystem changes across a 25‐year chronosequence. Ecological Applications 30(4), e02085. 10.1002/eap.2085. 31991504 PMC7423248

[r118] Owers CJ, Woodroffe CD, Mazumder D and Rogers K (2022) Carbon storage in coastal wetlands is related to elevation and how it changes over time. Estuarine, Coastal and Shelf Science 267, 107775. 10.1016/j.ecss.2022.107775.

[r119] Peralta G, van Duren L, Morris E and Bouma T (2008) Consequences of shoot density and stiffness for ecosystem engineering by benthic macrophytes in flow dominated areas: A hydrodynamic flume study. Marine Ecology Progress Series 368, 103–115. 10.3354/meps07574.

[r120] Pindilli E (2022) Ecosystem service co-benefits of wetland carbon management. In Krauss KW, Zhu Z, Stagg CL (eds.), Wetland Carbon and Environmental Management. Hoboken, NJ: American Geophysical Union and John Wiley and Sons, Inc., pp. 403–409.

[r121] Poffenbarger HJ, Needelman BA and Megonigal JP (2011) Salinity influence on methane emissions from tidal marshes. Wetlands 31(5), 831–842. 10.1007/s13157-011-0197-0.

[r122] Poulter B, Fluet-Chouinard E, Hugelius G, Koven C, Fatoyinbo L, Page SE, Rosentreter JA, Smart L, Taillie PJ, Thomas N, Zhang Z and Wijedasa L (2022) A review of global wetland carbon stocks and management challenges. In Krauss KW, Zhu Z, Stagg CL (eds.), Wetland Carbon and Environmental Management. Hoboken, NJ: American Geophysical Union and John Wiley and Sons, Inc., pp. 1–20

[r123] Qin G, Lu Z, Gan S, Zhang L, Wu J, Sanders CJ, He Z, Yu X, Zhang J, Zhou J, Ding R, Huang X, Chen H, He H, Yu M, Li H, and Wang F (2024) Fiddler crab bioturbation stimulates methane emissions in mangroves: Insights into microbial mechanisms. Soil Biology and Biogeochemistry 194, 109445. 10.1016/j.soilbio.2024.109445.

[r124] Ray GL (2007) Thin Layer Placement of Dredged Material on Coastal Wetlands: A Review of the Technical and Scientific Literature. Vicksburg, MS: U.S. Army Corp of Engineers, Army Engineer Research and Development Center, Environmental Laboratory, 8 pp.

[r125] Reithmaier GMS, Cabral A, Akhand A, Bogard MJ, Borges AV, Bouillon S, Burdige DJ, Call M, Chen N, Chen X, Cotovicz LC, Eagle MJ, Kristensen E, Kroeger KD, Lu Z, Maher DT, Pérez-Lloréns JL, Ray R, Taillardat P, Tamborski JJ, Upstill-Goddard RC, Wang F, Wang ZA, Xiao K, Yau YYY and Santos IR (2023) Carbonate chemistry and carbon sequestration driven by inorganic carbon outwelling from mangroves and saltmarshes. Nature Communications 14(1), 8196. 10.1038/s41467-023-44037-w. PMC10713528 38081846

[r126] Riddin T and Adams JB (2008) Influence of mouth status and water level on the macrophytes in a small temporarily open/closed estuary. Estuarine, Coastal and Shelf Science 79(1), 86–92. 10.1016/j.ecss.2008.03.010.

[r127] Rinke M, Maraun M and Scheu S (2022) Spatial and temporal variations in salt marsh microorganisms of the Wadden Sea. Ecology and Evolution 12(3), e8767. 10.1002/ece3.8767. 35356561 PMC8958242

[r128] Rogers K, Kelleway JJ, Saintilan N, Megonigal JP, Adams JB, Holmquist JR, Lu M, Schile-Beers L, Zawadzki A, Mazumder D and Woodroffe CD (2019a) Wetland carbon storage controlled by millennial-scale variation in relative sea-level rise. Nature 567(7746), 91–95. 10.1038/s41586-019-0951-7. 30842636

[r129] Rogers K, Kellway J and Saintilan N (2023a) The present, past and future of blue carbon. Cambridge Prisms: Coastal Futures 1, e30. 10.1017/cft.2023.17.

[r130] Rogers K, Lal KK, Asbridge EF and Dwyer PG (2023b) Coastal wetland rehabilitation first-pass prioritisation for blue carbon and associated co-benefits. Marine and Freshwater Research 74(3), 177–199. 10.1071/MF22014.

[r131] Rogers K, Macreadie PI, Kelleway JJ and Saintilan N (2019b) Blue carbon in coastal landscapes: A spatial framework for assessment of stocks and additionality. Sustainability Science 14(2), 453–467. 10.1007/s11625-018-0575-0.

[r132] Rogers K, Zawadzki A, Mogensen LA and Saintilan N (2022) Coastal wetland surface elevation change is dynamically related to accommodation space and influenced by sedimentation and sea-level rise over decadal timescales. Frontiers in Marine Science 9, 807588. 10.3389/fmars.2022.807588.

[r133] Rosentreter JA, Al‐Haj AN, Fulweiler RW and Williamson P (2021) Methane and nitrous oxide emissions complicate coastal blue carbon assessments. Global Biogeochemical Cycles 35(2), e2020GB006858. 10.1029/2020GB006858.

[r134] Rosentreter JA, Laruelle GG, Bange HW, Bianchi TS, Busecke JJM, Cai W-J, Eyre BD, Forbrich I, Kwon EY, Maavara T, Moosdorf N, Najjar RG, Sarma VVSS, Van Dam B and Regnier P (2023) Coastal vegetation and estuaries are collectively a greenhouse gas sink. Nature Climate Change 13(6), 579–587. 10.1038/s41558-023-01682-9.

[r135] Rovai AS, Twilley RR, Castañeda‐Moya E, Midway SR, Friess DA, Trettin CC, Bukoski JJ, Stovall AEL, Pagliosa PR, Fonseca AL, Mackenzie RA, Aslan A, Sasmito SD, Sillanpää M, Cole TG, Purbopuspito J, Warren MW, Murdiyarso D, Mofu W, Sharma S, Tinh PH and Riul P (2021) Macroecological patterns of forest structure and allometric scaling in mangrove forests. Global Ecology and Biogeography 30(5), 1000–1013. 10.1111/geb.13268.

[r136] Rovai AS, Twilley RR, Castañeda-Moya E, Riul P, Cifuentes-Jara M, Manrow-Villalobos M, Horta PA, Simonassi JC, Fonseca AL and Pagliosa PR (2018) Global controls on carbon storage in mangrove soils. Nature Climate Change 8(6), 534–538. 10.1038/s41558-018-0162-5.

[r137] Saintilan N, Kovalenko KE, Guntenspergen G, Rogers K, Lynch JC, Cahoon DR, Lovelock CE, Friess DA, Ashe E, Krauss KW, Cormier N, Spencer T, Adams J, Raw J, Ibanez C, Scarton F, Temmerman S, Meire P, Maris T, Thorne K, Brazner J, Chmura GL, Bowron T, Gamage VP, Cressman K, Endris C, Marconi C, Marcum P, St. Laurent K, Reay W, Raposa KB, Garwood JA and Khan N (2022) Constraints on the adjustment of tidal marshes to accelerating sea level rise. Science 377(6605), 523–527. 10.1126/science.abo7872. 35901146

[r138] Sanders-DeMott R, Eagle MJ, Kroeger KD, Wang F, Brooks TW, O-Keefe Suttles JA, Nick SK, Mann AG and Tang J (2022) Impoundment increases methane emissions in *Phragmites*-invaded coastal wetlands. Global Change Biology 28, 4539–4557. 10.1111/gcb.16217. 35616054

[r139] Sandi SG, Rodriguez JF, Saco PM, Saintilan N and Riccardi G (2021) Accelerated sea‐level rise limits vegetation capacity to sequester soil carbon in coastal wetlands: A study case in southeastern Australia. Earth’s Future 9(9), e2020EF001901. 10.1029/2020EF001901.

[r140] Santos IR, Burdige DJ, Jennerjahn TC, Bouillon S, Cabral A, Serrano O, Wernberg T, Filbee-Dexter K, Guimond JA and Tamborski JJ (2021) The renaissance of Odum’s outwelling hypothesis in ‘Blue Carbon’ science. Estuarine, Coastal and Shelf Science 255, 107361. 10.1016/j.ecss.2021.107361.

[r141] Santos IR, Maher DT, Larkin R, Webb JR and Sanders CJ (2019) Carbon outwelling and outgassing vs. burial in an estuarine tidal creek surrounded by mangrove and saltmarsh wetlands. Limnology and Oceanography 64(3), 996–1013. 10.1002/lno.11090.

[r142] Sasmito SD, Taillardat P, Clendenning JN, Cameron C, Friess DA, Murdiyarso D and Hutley LB (2019) Effect of land‐use and land‐cover change on mangrove blue carbon: A systematic review. Global Change Biology 25(12), 4291–4302. 10.1111/gcb.14774. 31456276

[r143] Schieder NW, Walters DC and Kirwan ML (2018) Massive upland to wetland conversion compensated for historical marsh loss in Chesapeake Bay, USA. Estuaries and Coasts 41(4), 940–951. 10.1007/s12237-017-0336-9.

[r144] Schoolmaster DR, Stagg CL, Creamer C, Laurenzano C, Ward EJ, Waldrop MP, Baustian MM, Aw T, Merino S, Villani R and Scott L (2022) A model of the spatiotemporal dynamics of soil carbon following coastal wetland loss applied to a Louisiana salt marsh in the Mississippi River deltaic plain. Journal of Geophysical Research: Biogeosciences 127(6), e2022JG006807. 10.1029/2022JG006807.

[r145] Schutte CA, Moore WS, Wilson AM and Joye SB (2020) Groundwater‐driven methane export reduces salt marsh blue carbon potential. Global Biogeochemical Cycles 34(10), e2020GB006587. 10.1029/2020GB006587.

[r146] Segarra KEA, Comerford C, Slaughter J and Joye SB (2013) Impact of electron acceptor availability on the anaerobic oxidation of methane in coastal freshwater and brackish wetland sediments. Geochimica et Cosmochimica Acta 115, 15–30. 10.1016/j.gca.2013.03.029.

[r147] Shahan J, Chu H, Windham‐Myers L, Matsumura M, Carlin J, Eichelmann E, Stuart‐Haentjens E, Bergamaschi B, Nakatsuka K, Sturtevant C and Oikawa P (2022) Combining eddy covariance and chamber methods to better constrain CO_2_ and CH_4_ fluxes across a heterogeneous restored tidal wetland. Journal of Geophysical Research: Biogeosciences 127(9), e2022JG007112. 10.1029/2022JG007112.

[r148] Sidik F, Fernanda Adame M and Lovelock CE (2019) Carbon sequestration and fluxes of restored mangroves in abandoned aquaculture ponds. Journal of the Indian Ocean Region 15(2), 177–192. 10.1080/19480881.2019.1605659.

[r149] Sinclair EA, Sherman CDH, Statton J, Copeland C, Matthews A, Waycott M, van Dijk K, Vergés A, Kajlich L, McLeod IM and Kendrick GA (2021) Advances in approaches to seagrass restoration in Australia. Ecological Management & Restoration 22(1), 10–21. 10.1111/emr.12452.

[r150] Sleeter BM, Frid L, Rayfield B, Daniel C, Zhu Z and Marvin DC (2022) Operational assessment tool for forest carbon dynamics for the United States: A new spatially explicit approach linking the LUCAS and CBM-CFS3 models. Carbon Balance and Management 17(1), 1. 10.1186/s13021-022-00201-1. 35107646 PMC8811977

[r151] Sleeter BM, Liu J, Daniel C, Rayfield B, Sherba J, Hawbaker TJ, Zhu Z, Selmants PC and Loveland TR (2018) Effects of contemporary land-use and land-cover change on the carbon balance of terrestrial ecosystems in the United States. Environmental Research Letters 13(4), 045006. 10.1088/1748-9326/aab540.

[r152] Smith S (2024) The effects of *Sesarma reticulatum* (L.) herbivory and sea level rise on creek expansion in Cape Cod salt marshes. Continental Shelf Research 272, 105146. 10.1016/j.csr.2023.105146.

[r153] Stagg, CL and Mendelssohn IA (2010) Restoring ecological function to a submerged salt marsh. Restoration Ecology 18, 10–17. 10.1111/j.1526-100X.2010.00718.x.

[r154] Stewart-Sinclair PJ, Purandare J, Bayraktarov E, Waltham N, Reeves S, Statton J, Sinclair EA, Brown BM, Shribman ZI and Lovelock CE (2020) Blue restoration – Building confidence and overcoming barriers. Frontiers in Marine Science 7, 541700. 10.3389/fmars.2020.541700.

[r155] Surgeon Rogers T, Kroeger KD, Gonneea ME, Abdul-Aziz O, Tang J and Moseman-Valtierra S (2019) Blue carbon as a tool to support coastal management and restoration: Bringing wetlands to market case study. In Windham-Myers L, Crooks S, Troxler T (eds.), A Blue Carbon Primer: The State of Coastal Wetland Carbon Science, Practice and Policy. Boca Raton, FL: CRC Press, Taylor & Francis Group, pp. 353–365.

[r156] Sweet WV, Hamlington BD, Kopp RE, Weaver CP, Barnard PL and Bekaert D (2022) Global and Regional Sea Level Rise Scenarios for the United States: Updated Mean Projections and Extreme Water Level Probabilities Along U.S. Coastlines (No. NOS 01). Silver Spring, MD: National Oceanic and Atmospheric Administration. Available at https://oceanservice.noaa.gov/hazards/sealevelrise/noaa-nostechrpt01-global-regional-SLR-scenarios-US.pdf.

[r157] Taillardat P, Thompson BS, Garneau M, Trottier K and Friess DA (2020) Climate change mitigation potential of wetlands and the cost-effectiveness of their restoration. Interface Focus 10(5), 20190129. 10.1098/rsfs.2019.0129. 32832065 PMC7435041

[r158] Tangen BA and Bansal S (2022) Prairie wetlands as sources or sinks of nitrous oxide: Effects of land use and hydrology. Agricultural and Forest Meteorology 320, 108968. 10.1016/j.agrformet.2022.108968.

[r159] Thorne K, Jones S, Freeman C, Buffington K, Janousek C and Guntenspergen G (2022) Atmospheric river storm flooding influences tidal marsh elevation building processes. Journal of Geophysical Research: Biogeosciences 127(3), e2021JG006592. 10.1029/2021JG006592.

[r160] Thorslund J, Jarsjo J, Jaramillo F, Jawitz JW, Manzoni S, Basu NB, Chalov SR, Cohen MJ, Creed IF, Goldenberg R, Hylin A, Kalantari Z, Koussis AD, Lyon SW, Mazi K, Mard J, Persson K, Pietro J, Prieto C, Quin A, Van Meter K and Destouni G (2017) Wetlands as large-scale nature-based solutions: Status and challenges for research, engineering and management. Ecological Engineering 108, 489–497. 10.1016/j.ecoleng.2017.07.012.

[r161] Turschwell MP, Connolly RM, Dunic JC, Sievers M, Buelow CA, Pearson RM, Tulloch VJD, Côté IM, Unsworth RKF, Collier CJ and Brown CJ (2021) Anthropogenic pressures and life history predict trajectories of seagrass meadow extent at a global scale. Proceedings of the National Academy of Sciences 118(45), e2110802118. 10.1073/pnas.2110802118. PMC8609331 34725160

[r162] Twomey AJ, Nunez K, Carr JA, Crooks S, Friess DA, Glamore W, Orr M, Reef R, Rogers K, Waltham NJ and Lovelock CE (2024) Planning hydrological restoration of coastal wetlands: Key model considerations and solutions. Science of the Total Environment 915, 169881. 10.1016/j.scitotenv.2024.169881. 38190895

[r163] United Nations Environment Programme (UNEP) (2021) Becoming #GenerationRestoration: Ecosystem restoration for people, nature and climate. Nairobi: UNEP.

[r164] United Nations Environment Programme (UNEP) and International Union for Conservation of Nature (IUCN) (2021) Nature-based solutions for climate change mitigation. Nairobi and Gland: UNEP.

[r165] United Nations Framework Commission on Climate Change (UNFCCC) (2015) Adoption of the Paris Agreement (No. FCCC/CP/2015/L.9/Rev.1). New York, NY: United Nations.

[r166] Vahsen ML, Todd‐Brown KEO, Hicks J, Pilyugin SS, Morris JT and Holmquist JR (2024) Cohort marsh equilibrium model (CMEM): History, mathematics, and implementation. Journal of Geophysical Research: Biogeosciences 129(4), e2023JG007823. 10.1029/2023JG007823.

[r167] Valach AC, Kasak K, Hemes KS, Szutu D, Verfaillie J and Baldocchi DD (2021) Carbon flux trajectories and site conditions from restored impounded marshes in the Sacramento‐San Joaquin Delta. In Krauss KW, Zhu Z, and Stagg CL (eds), Geophysical Monograph Series, 1st ed. Hoboken, NJ American Geophysical Union and John Wiley and Sons, Inc., pp. 247–f. 10.1002/9781119639305.ch13.

[r168] van Bijsterveldt CEJ, Debrot AO, Bouma TJ, Maulana MB, Pribadi R, Schop J, Tonneijck FH and van Wesenbeeck BK (2022) To plant or not to plant: When can planting facilitate mangrove restoration? Frontiers in Environmental Science 9, 690011. 10.3389/fenvs.2021.690011.

[r169] van Katwijk MM, Thorhaug A, Marbà N, Orth RJ, Duarte CM, Kendrick GA, Althuizen IHJ, Balestri E, Bernard G, Cambridge ML, Cunha A, Durance C, Giesen W, Han Q, Hosokawa S, Kiswara W, Komatsu T, Lardicci C, Lee K, Meinesz A, Nakaoka M, O’Brien KR, Paling EI, Pickerell C, Ransijn AMA and Verduin JJ (2016) Global analysis of seagrass restoration: The importance of large‐scale planting. Journal of Applied Ecology 53(2), 567–578. 10.1111/1365-2664.12562.

[r170] Vanderklift MA, Doropoulos C, Gorman D, Leal I, Minne AJP, Statton J, Steven ADL and Wernberg T (2020) Using propagules to restore coastal marine ecosystems. Frontiers in Marine Science 7, 724. 10.3389/fmars.2020.00724.

[r171] Vegh T, Pendleton L, Murray B, Troxler T, Zhang K, Castañeda-Moya E, Guannel G and Sutton-Grier A (2019) Ecosystem services and economic valuation: Co-benefits of coastal wetlands. In Windham-Myers L, Crooks S, Troxler T (eds.) A Blue Carbon Primer: The State of Coastal Wetland Carbon Science, Practice and Policy. Boca Raton, FL: CRC Press, Taylor & Francis Group, pp. 249–266.

[r172] Villa JA, Ju Y, Stephen T, Rey‐Sanchez C, Wrighton KC and Bohrer G (2020) Plant‐mediated methane transport in emergent and floating‐leaved species of a temperate freshwater mineral‐soil wetland. Limnology and Oceanography 65(7), 1635–1650. 10.1002/lno.11467.

[r173] Vroom RJE, van den Berg M, Pangala SR, van der Scheer OE and Sorrell BK (2022) Physiological processes affecting methane transport by wetland vegetation – A review. Aquatic Botany 182, 103547. 10.1016/j.aquabot.2022.103547.

[r174] Walker AK and Campbell J (2010) Marine fungal diversity: A comparison of natural and created salt marshes of the north-central Gulf of Mexico. Mycologia 102(3), 513–521. 10.3852/09-132. 20524584

[r175] Wang H, Dai Z, Trettin CC, Krauss KW, Noe GB, Burton AJ, Stagg CL and Ward EJ (2022a) Modeling impacts of drought‐induced salinity intrusion on carbon dynamics in tidal freshwater forested wetlands. Ecological Applications 32(8), e2700. 10.1002/eap.2700. 35751513

[r176] Wang H, Ho M, Flanagan N and Richardson CJ (2021) The effects of hydrological management on methane emissions from southeastern shrub bogs of the USA. Wetlands 41(7), 87. 10.1007/s13157-021-01486-7.

[r177] Wang H, Krauss KW, Noe GB, Dai Z and Trettin CC (2023) Soil salinity and water level interact to generate tipping points in low salinity tidal wetlands responding to climate change. Estuaries and Coasts 46, 1808–1828. 10.1007/s12237-023-01243-5.

[r178] Wang Z, Sadat-Noori M and Glamore W (2022b) Groundwater discharge drives water quality and greenhouse gas emissions in a tidal wetland. Water Science and Engineering 15(2), 141–151. 10.1016/j.wse.2022.02.005.

[r179] Warren RS, Fell PE, Rozsa R, Brawley AH, Orsted AC, Olson ET, Swamy V and Niering WA (2002) Salt marsh restoration in Connecticut: 20 years of science and management. Restoration Ecology 10(3), 497–513. 10.1046/j.1526-100X.2002.01031.x.

[r180] Windham-Myers L, Oikawa P, Deverel S, Chapple D, Drexler J and Stern D (2023) Carbon sequestration and subsidence reversal in the Sacramento-San Joaquin Delta and Suisun Bay: Management opportunities for climate mitigation and adaptation. San Francisco Estuary and Watershed Science 20(4), 1–29. 10.15447/sfews.2023v20iss4art7.

[r181] Wodehouse DCJ and Rayment MB (2019) Mangrove area and propagule number planting targets produce sub-optimal rehabilitation and afforestation outcomes. Estuarine, Coastal and Shelf Science 222, 91–102. 10.1016/j.ecss.2019.04.003.

[r182] Wollenberg JT, Ollerhead J and Chmura GL (2018) Rapid carbon accumulation following managed realignment on the Bay of Fundy. PLOS ONE 13(3), e0193930. 10.1371/journal.pone.0193930. 29561874 PMC5862474

[r183] Woo I, Davis M, De La Cruz S, Windham-Myers L, Drexler J, Byrd K, Stuart-Haëntjens E, Anderson F, Bergamaschi B, Nakai G, Ellings C and Hodgson S (2022) Carbon flux, storage, and wildlife co-benefits in a restoring estuary: Case study at the Nisqually River Delta, Washington. In Krauss KW, Zhu Z, Stagg CL (eds.). Wetland Carbon and Environmental Management, Hoboken, NJ: American Geophysical Union and John Wiley and Sons, Inc., pp. 105–125.

[r184] Woodroffe CD (2019) The morphology and development of coastal wetlands in the tropics. In Perillo GME, Wolanski E, Cahoon DR, Hopkinson CS (eds.). Coastal Wetlands: An Integrated Ecosystem Approach. Amsterdam, The Netherlands: Elsevier B.V., pp. 79–103. 10.1016/B978-0-444-63893-9.00002-2.

[r185] Worthington TA, Spalding M, Landis E, Maxwell TL, Navarro A, Smart LS and Murray NJ (2024) The distribution of global tidal marshes from Earth observation data. Global Ecology and Biogeography 33, e13852. 10.1111/geb.13852

[r186] Wortley L, Hero J and Howes M (2013) Evaluating ecological restoration success: A review of the literature. Restoration Ecology 21(5), 537–543. 10.1111/rec.12028.

[r187] Wylie L, Sutton-Grier AE and Moore A (2016) Keys to successful blue carbon projects: Lessons learned from global case studies. Marine Policy 65, 76–84. 10.1016/j.marpol.2015.12.020.

[r188] Xiao K, Wilson AM, Li H, Santos IR, Tamborski J, Smith E, Lang SQ, Zheng C, Luo X, Lu M and Correa RE (2021) Large CO_2_ release and tidal flushing in salt marsh crab burrows reduce the potential for blue carbon sequestration. Limnology and Oceanography 66(1), 14–29. 10.1002/lno.11582.

[r189] Yando ES, Jones SF, James WR, Colombano DD, Montemayor DI, Nolte S, Raw JL, Ziegler SL, Chen L, Daffonchio D, Fusi M, Rogers K and Sergienko L (2023) An integrative salt marsh conceptual framework for global comparisons. Limnology and Oceanography Letters 8, 830–849. 10.1002/lol2.10346.

[r190] Yau YYY, Xin P, Chen X, Zhan L, Call M, Conrad SR, Sanders CJ, Li L, Du J and Santos IR (2022) Alkalinity export to the ocean is a major carbon sequestration mechanism in a macrotidal saltmarsh. Limnology and Oceanography 67(S2), S158–S170. 10.1002/lno.12155.

[r191] Yuan L, Liu D, Tian B, Yuan X, Bo S, Ma Q, Wu W, Zhao Z, Zhang L and Keesing JK (2022) A solution for restoration of critical wetlands and waterbird habitats in coastal deltaic systems. Journal of Environmental Management 302, 113996. 10.1016/j.jenvman.2021.113996. 34717102

[r192] Zamith LR and Scarano FR (2010) Restoration of a coastal swamp forest in southeast Brazil. Wetlands Ecology and Management 18, 435–448. 10.1007/s11273-010-9177-z.

[r193] Zhu Q, Chen J, Wu L, Huang Y, Shao C, Dong G, Xu Z and Li X (2024) Changes in albedo and its radiative forcing of grasslands in East Asia drylands. Ecological Processes 13(1), 17. 10.1186/s13717-024-00493-w.

