# Supplementary material for: When and where can coastal wetland restoration increase carbon sequestration as a natural climate solution?
Source: Camb Prism Coast Futur. 2024 Oct 11;2:e13. doi: 10.1017/cft.2024.14 (PMC12337610; doi:10.1017/cft.2024.14)
Supplement: Peer review history

# Editor report: Recommendation: When and where can coastal wetland restoration increase carbon sequestration as a natural climate solution? — R0/PR2

**DOI:** 10.1017/cft.2024.14.pr2

**Affiliations:** Second Institute of Oceanography Ministry of Natural Resources, China

No accompanying comment.

# Editor report: Decision: When and where can coastal wetland restoration increase carbon sequestration as a natural climate solution? — R0/PR3

**DOI:** 10.1017/cft.2024.14.pr3

**Affiliations:** Geography, Cambridge University, United Kingdom of Great Britain and Northern Ireland

No accompanying comment.

# Editor report: Recommendation: When and where can coastal wetland restoration increase carbon sequestration as a natural climate solution? — R1/PR5

**DOI:** 10.1017/cft.2024.14.pr5

**Affiliations:** Second Institute of Oceanography Ministry of Natural Resources, China

No accompanying comment.

# Editor report: Decision: When and where can coastal wetland restoration increase carbon sequestration as a natural climate solution? — R1/PR6

**DOI:** 10.1017/cft.2024.14.pr6

**Affiliations:** Geography, Cambridge University, United Kingdom of Great Britain and Northern Ireland

No accompanying comment.
